# Cryo-EM structure of the human MLL1 core complex bound to the nucleosome

**DOI:** 10.1038/s41467-019-13550-2

**Published:** 2019-12-05

**Authors:** Sang Ho Park, Alex Ayoub, Young-Tae Lee, Jing Xu, Hanseong Kim, Wei Zheng, Biao Zhang, Liang Sha, Sojin An, Yang Zhang, Michael A. Cianfrocco, Min Su, Yali Dou, Uhn-Soo Cho

**Affiliations:** 1 Department of Biological Chemistry, University of Michigan, Ann Arbor, Michigan 48109 USA; 2 Department of Pathology, University of Michigan, Ann Arbor, Michigan 48109 USA; 3 Computational Medicine and Bioinformatics, University of Michigan, Ann Arbor, Michigan 48109 USA; 4 Life Sciences Institute, University of Michigan, Ann Arbor, Michigan 48109 USA

**Keywords:** Cryoelectron microscopy, Epigenetics

## Abstract

Mixed lineage leukemia (MLL) family histone methyltransferases are enzymes that deposit histone H3 Lys4 (K4) mono-/di-/tri-methylation and regulate gene expression in mammals. Despite extensive structural and biochemical studies, the molecular mechanisms whereby the MLL complexes recognize histone H3K4 within nucleosome core particles (NCPs) remain unclear. Here we report the single-particle cryo-electron microscopy (cryo-EM) structure of the NCP-bound human MLL1 core complex. We show that the MLL1 core complex anchors to the NCP via the conserved RbBP5 and ASH2L, which interact extensively with nucleosomal DNA and the surface close to the N-terminal tail of histone H4. Concurrent interactions of RbBP5 and ASH2L with the NCP uniquely align the catalytic MLL1^SET^ domain at the nucleosome dyad, thereby facilitating symmetrical access to both H3K4 substrates within the NCP. Our study sheds light on how the MLL1 complex engages chromatin and how chromatin binding promotes MLL1 tri-methylation activity.

## Abstract (web summary)

MLL family histone methyltransferases deposit histone H3 Lys4 mono-/di-/tri-methylation and regulate gene expression in mammals. Here the authors report the single-particle cryo-EM structure of the NCP-bound human MLL1 core complex, shedding light on how the MLL1 complex engages chromatin and how chromatin binding promotes MLL1 tri-methylation activity.

## Introduction

The nucleosome core particle (NCP), consisting of an octameric core of histone proteins (two of each H2A, H2B, H3, and H4) and 146 base pairs of genomic DNA, represents the first level of eukaryotic DNA packaging^1^. It is further organized into higher-order chromatin structures. Cell-specific transcription program, in large part, is governed by chromatin accessibility, which is actively regulated by histone-modifying enzymes and ATP-dependent chromatin-remodeling complexes. In recent years, X-ray crystallography and single-particle cryo-electron microscopy (cryo-EM) studies have shed light on how these chromatin-associating complexes interact with the NCP for respective physiological functions. Most, if not all, chromatin complexes engage the “acidic-patch” region of the NCP through variations of an arginine-finger motif^2–5^, highlighting common features among chromatin-interacting protein complexes. It remains unclear whether the recognition mode of the NCP is universal for chromatin-interacting complexes.

Among histone posttranslational modifications, histone H3 lysine (K) 4 methylation (H3K4me) is exquisitely regulated at key transcription regulatory regions^6^. In particular, H3K4me3 is highly enriched at the transcriptionally active gene promoters while H3K4me1 is a prevalent mark at poised or active distal enhancers^6^. Distinct H3K4 methylation states can be recognized by well-defined chromatin reader modules that lead to specific transcription outcomes^7^. High levels of H3K4me3, often at open-chromatin regions, is crucial for recruitment of the basic transcription machinery^8^, ATP-dependent chromatin-remodeling complexes^9^, and histone acetyltransferases, and plays direct roles in transcription activation^10^. Dynamic changes in broad H3K4me3 domains have been described for maternal-to-zygotic transition in early development^11^ and is associated with increased transcription activity at tumor suppressor genes^12,13^. Thus, understanding how specific H3K4me state is regulated is important for deciphering the mechanism underlying the physiological and pathological processes.

Mixed lineage leukemia (MLL) family enzymes, including MLL1-4/KMT2A-2D, SET1A/KMT2F, and SET1B/KMT2G, are the major histone lysine 4 (K4) methyltransferases in mammals^6^. The MLL family enzymes share an evolutionarily conserved catalytic Su(Var)3–9, Enhancer of Zeste, Trithorax (SET) domain^14^ as well as four highly conserved SET-interacting proteins, i.e., RbBP5 (retinoblastoma-binding protein 5), ASH2L (Absent, small, homeotic disks-2-like), WDR5 (WD40 repeat-containing protein 5), and DPY30 (DumPY protein 30)^6^. Aberrant expression and recurrent mutations of the MLL family enzymes, as well as its non-catalytic core components, e.g., ASH2L and RbBP5, have been identified in a wide spectrum of human malignancies^15–18^ and a variety of congenital human syndromes including Kabuki, Wiedemann–Steiner, and Kleefstra spectrum syndromes^6^, indicative the importance of this family of protein complexes in normal cellular functions and in development.

Biochemical studies show that the core components of the MLL1 complex are able to enhance the MLL1^SET^ activity for H3K4me1 and H3K4me2 by ~600-fold^19^. Mechanisms underlying this stimulation has been elegantly demonstrated in several structural studies of the human MLL1/3^SET^-ASH2L^SPRY^-RbBP5^330–375^ subcomplex^16^, the homologous yeast SET1 complexes^20,21^, as well as individual MLL1 core components^22–24^. However, these previous structures of the MLL SET domains^16,25,26^ were determined with either no substrate or H3 peptide as the substrate. It remains unclear how the MLL1 complex interacts and catalyzes H3K4 methylation within the physiological substrate, i.e., NCP. More importantly, it remains to be determined how MLL1 activity, especially the tri-methylation activity, is regulated on chromatin.

Here we report the single-particle cryo-EM structure of the human MLL1 core complex bound to the NCP. It not only reveals the overall architecture of the human MLL1 complex with full-length core components, but also illustrates how the MLL1 core complex engages the chromatin. Importantly, we show that the MLL1 core complex docks on the NCP through concurrent interactions of ASH2L/RbBP5 with nucleosomal DNA and histone H4. This unique configuration aligns the catalytic MLL1^SET^ domain at the nucleosome dyad, which allows the symmetric access to both H3K4 substrates. Our structure sheds light on how the MLL1 complex binds to the chromatin and how its activity for H3K4me3 is regulated.

## Results

### Architecture of the NCP-bound human MLL1 core complex

**Fig. 1:**
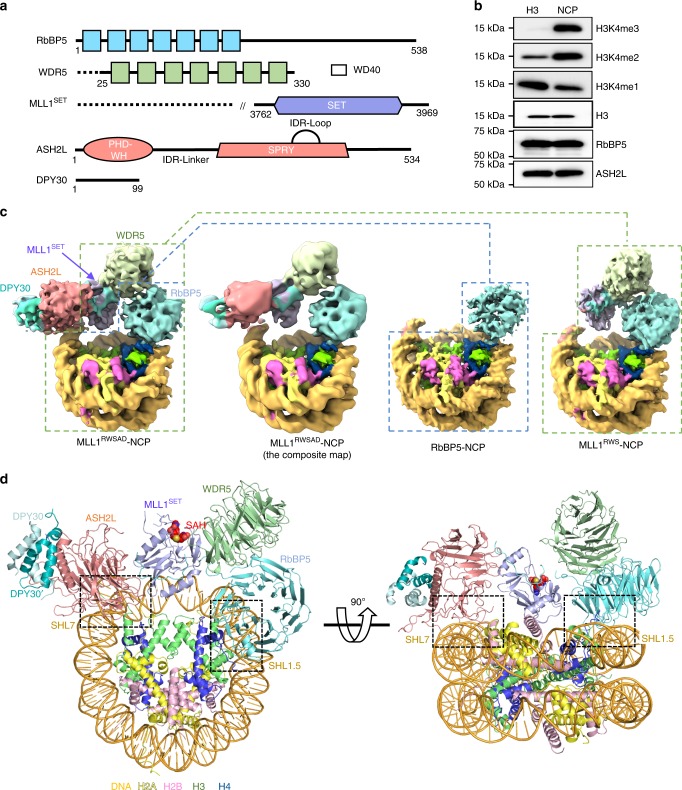
Cryo-EM structure of the MLL1^RWSAD^-NCP complex. **a** Schematic domain architectures for the core components of the human MLL1 complex used in the cryo-EM study. **b** Immunoblot to detect H3K4 methylation in the in vitro histone methyltransferase assay. Antibodies indicated on right. The substrates were free recombinant histone H3 (left) and the NCP (right), respectively. Immunoblots for unmodified H3 as well as RbBP5 and ASH2L included as controls. **c** Cryo-EM 3D reconstruction of the MLL1^RWSAD^-NCP complex. The composite map of MLL1^RWSAD^-NCP was locally filtered to the estimated resolution. The subcomplexes, i.e., RbBP5-NCP and MLL1^RWS^-NCP, shown in dashed boxes. **d** Top (left) and front (right) views of the MLL1^RWSAD^-NCP structure. The *S*-adenosyl-l-homocysteine (SAH) was represented as a sphere (red) and the MLL1 core components shown in cartoon representation (RbBP5: cyan, WDR5: green, MLL1^SET^: slate, ASH2L: pink, and DPY30 dimer: cerulean and teal). Widom 601 DNA and four histones were colored as indicated on the bottom. Two black dashed squares highlighted the nucleosome contact points near SHL1.5 and SHL7 by MLL1^RWSAD^. Illustrations of the protein structure and cryo-EM maps used in all figures were generated with PyMOL (Delano Scientific, LLC) and Chimera^67^/ChimeraX^70^.

A recombinant human MLL1 core complex (MLL1^RWSAD^) containing RbBP5 (residues 1–538), WDR5 (residues 25–330), MLL1^SET^ (residues 3762–3969), ASH2L (residues 1–534), and DPY30 (residues 1–99) was reconstituted in vitro (Fig. 1a and Supplementary Fig. 1a). An electrophoretic mobility shift assay (EMSA) demonstrated that MLL1^RWSAD^ binds to the NCP with modest affinity (Supplementary Fig. 1b, c and data not shown). We found that this complex showed markedly enhanced activity for higher methylation states (i.e., H3K4me2 and H3K4me3) when the NCP was used as a substrate (Fig. 1b and Supplementary Fig. 1d). To elucidate the mechanisms underlying this interaction, we determined the single-particle cryo-EM structure of reconstituted MLL1^RWSAD^ bound to the recombinant NCP. The cryo-EM structure of MLL1^RWSAD^-NCP was determined at a resolution of 6.2 Å (Fig. 1c, Supplementary Figs. 2 and 3a, and Supplementary Table 1). A composite map of MLL1^RWSAD^-NCP was generated after local filtering to the estimated resolution to avoid over-interpretation (Fig. 1c and Supplementary Fig. 2). In parallel, the cryo-EM maps of RbBP5-NCP and RbBP5-WDR5-MLL1^SET^ (MLL1^RWS^)-NCP subcomplexes were derived from the MLL1^RWSAD^-NCP dataset and reconstructed at 4.2 Å and 4.5 Å resolution, respectively (Supplementary Figs. 2 and 3b–d, and Methods). The model structure of the MLL1^RWSAD^-NCP complex (Fig. 1d) was built by rigid-body fitting and real space refinement using crystal structures of mouse RbBP5 (PDB ID: 5OV3)^27^, human WDR5 (PDB ID: 2H14)^28^, human MLL1^SET^-ASH2L^SPRY^-RbBP5^330–375^ (PDB ID: 5F6L)^16^, a DPY30 dimer (PDB ID: 6E2H)^29^, and the 601-NCP (PDB ID: 3MVD)^3^.

The overall architecture of the MLL1^RWSAD^-NCP complex revealed that MLL1^RWSAD^ anchors at the edge of the NCP through two core components, RbBP5 and ASH2L (Fig. 1d). Within the NCP, we observed that DNA superhelical location 7 (SHL7) and SHL1.5, together with H4 N-terminal tail, were involved in the interaction with MLL1^RWSAD^ (Fig. 1d). Notably, domains in the MLL1^RWSAD^-NCP complex were found to be dynamically associated with each other and show multiple conformations (Supplementary Fig. 3e and Supplementary Movie 1). However, the overall architecture was conserved with respect to all subclasses of the MLL1^RWSAD^-NCP structures (Supplementary Fig. 3e and Supplementary Movie 1). Distinct from many of the previously reported NCP-recognizing proteins or protein complexes^30^, the MLL1 core complex did not interact with the acidic-patch region of the NCP.

### RbBP5 binds the NCP through both DNA and histone H4 tail

**Fig. 2:**
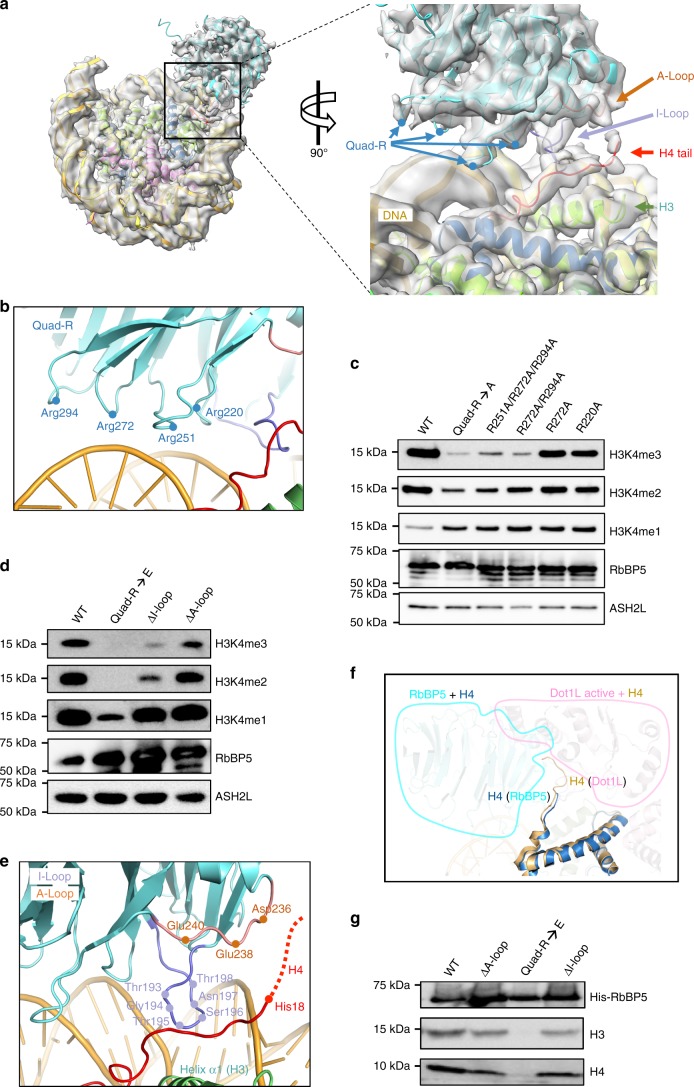
RbBP5 interaction with the NCP. **a** The cryo-EM structure of the RbBP5-NCP subcomplex (4.2 Å). The interaction interface was enlarged and shown on right. Insertion (I)-loop, Anchoring (A)-loop, and Quad-R of RbBP5, as well as the H4 tail highlighted in purple, orange, blue, and red, respectively. Histone H3 shown in green. **b** Interaction of Quad-R, as indicated, with DNA backbone. Red line, histone H4 tail. **c** Immunoblot to detect in vitro histone methyltransferase activity with the NCP as the substrate. The MLL1^RWSAD^ complex reconstituted with wild-type and Quad-R-mutated RbBP5 indicated on top. **d** Immunoblot to detect in vitro histone methyltransferase activity with the NCP as the substrate. The MLL1^RWSAD^ complex reconstituted with RbBP5 wild-type and deletion mutant proteins indicated on top. **e** The interface between RbBP5 and the H4 tail. Key residues on RbBP5 I-/A-loops indicated. The H4 tail (His18 to core) represented by a red line and the extended tail beyond His18 represented by a dash line. **f** Structural superposition of the H4 tails upon RbBP5 (cyan) and Dot1L (PDB ID: 6NJ9)^31^ binding. The RbBP5 and Dot1L at the interfaces enclosed by the blue and pink outlines, respectively. **g** In vitro pull-down assay for RbBP5 and the NCP. Ni-NTA-bound fractions were shown and His-tagged wild-type or mutant RbBP5 proteins shown on top. Immunoblot for H3 used to detect the NCP in the bound fraction. Immunoblot for H4 used as a control.

Within the MLL1^RWSAD^-NCP complex, the RbBP5-NCP interfaces were found to be less dynamic. The sub-population particles of RbBP5-NCP from the MLL1^RWSAD^-NCP dataset were resolved at a resolution of 4.2 Å (Fig. 2a and Supplementary Figs. 2 and 3b). The regions of mouse RbBP5 in model fitting shared 100% sequence identity with human RbBP5 (Supplementary Fig. 4a). The structure showed that RbBP5 bound to the NCP by simultaneously engaging DNA (SHL1.5) and histone H4 N-terminal tail. The interactions involved six consecutive loops emanating from the WD40 repeats of RbBP5 (Fig. 2a). Characteristic features of RbBP5 (e.g., unique helix, anchoring loop, and insertion loop) were well matched with the cryo-EM map of RbBP5-NCP subcomplex (Supplementary Fig. 4b). Notably, RbBP5 interacted with DNA SHL1.5 through four positively charged arginine residues (Quad-R) located in the β18–β19 (R220), β20–β21 (R251), β22–β23 (R272), and β24–β25 (R294) loops, respectively, which made electrostatic interactions with the DNA phosphate backbone (Fig. 2b). Disruption of the RbBP5-NCP interaction significantly reduced the activity of the MLL1 core complex. Mutations of the Quad-R residues to alanine (A) led to a reduction in H3K4me3 and to a lesser degree, H3K4me2 (Fig. 2c). The effect was more pronounced when the Quad-R residues were mutated to glutamic acid (E) (Fig. 2d). Systematic alteration of three, two or one arginine residue(s) in Quad-R revealed that at least two arginine residues were required for optimal H3K4me3 activity (Fig. 2c).

The second RbBP5-NCP interface includes two loops, an insertion loop (β16–β17 loop, referred to herein as I-loop) and an anchoring loop (β19–β20 loop, referred to herein as A-loop), of RbBP5 (Fig. 2a,e and Supplementary Figs. 4b and 5a). Both the I- and A-loops are evolutionarily conserved in higher eukaryotes (Supplementary Fig. 5b). The I-loop was positioned between the N-terminal tail of histone H4 and nucleosomal DNA (Fig. 2e). The A-loop run parallel to the H4 tail, which was positioned between the I-/A-loops of RbBP5 and the helix α1 (Leu65–Asp77) of histone H3 (Fig. 2e). This H4 tail-mediated nucleosome recognition of RbBP5 resembles that of the active-state DOT1L and SNF2h (Fig. 2f)^31,32^. Similar to Quad-R, deletion of the I-loop, and to a lesser degree the A-loop, reduced the activity of the MLL1 core complex for H3K4me3 and H3K4me2 (Fig. 2d). Importantly, RbBP5-NCP interaction is only required for MLL1 activity on the NCP (Fig. 2d). Mutations in Quad-R, the I-loop, and the A-loop had no effects on the mono-, di-, and tri-methylation of free H3 (Supplementary Fig. 5c). Among the RbBP5-NCP interactions, Quad-R was observed to be the main contributor to NCP binding. The mutation of Quad-R significantly reduced RbBP5 binding to the NCP, whereas deletion of the I- and A-loops had only a modest effect on NCP binding (Fig. 2g).

### Structure of a WDR5-MLL1^SET^–ASH2L^SPRY^ subcomplex

**Fig. 3:**
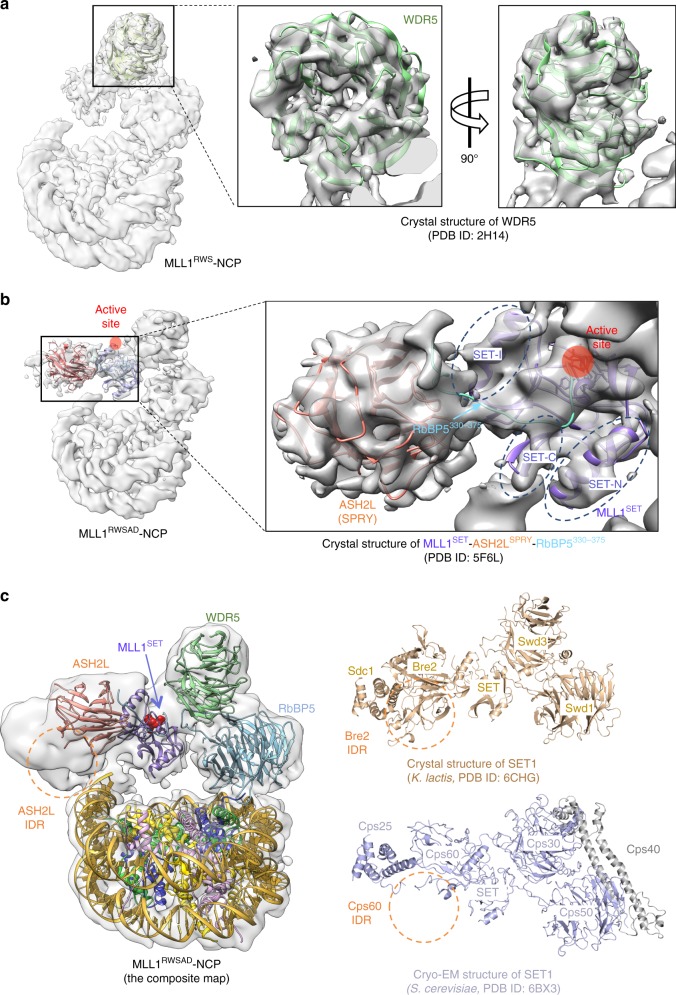
The WDR5-MLL1^SET^-ASH2L^SPRY^-NCP subcomplex. **a** Rigid-body fitting of human WDR5 crystal structure (PDB ID: 2H14)^28^ into the cryo-EM map of MLL1^RWS^-NCP. Secondary structures of WDR5 were shown in green. **b** Rigid-body fitting of MLL1^SET^ and ASH2L^SPRY^ into the Cryo-EM map of MLL1^RWSAD^-NCP. The MLL1^SET^-RbBP5^330–375^-ASH2L^SPRY^ crystal structure (PDB ID: 5F6L)^16^ was used. Characteristic secondary structures of MLL1^SET^ (SET-I, SET-C and SET-N) were shown within black dashed circles. The catalytic active site represented by red sphere. **c**, Comparison of human MLL1-NCP structure with yeast SET1 crystal structure (*K. lactis*, dark goldenrod, PDB ID: 6CHG, right top)^20^ and cryo-EM structure (*S. cerevisiae*, light slate blue, PDB ID: 6BX3, right bottom)^21^. Orange circles indicated IDR regions of ASH2L (human), Bre2 (*K. lactis*), and Cps60 (*S. cerevisiae*). An extra domain in *S. cerevisiae* SET1 complex, Cps40, colored grey.

To resolve the structural organization of the WDR5, MLL1^SET^, and ASH2L^SPRY^ subcomplex, we reconstructed the MLL1^RWS^-NCP subcomplex (Fig. 1c, Supplementary Fig. 2, and Methods) and successfully docked the crystal structures of human WDR5 (PDB ID: 2H14)^28^ and MLL1^SET^-ASH2L^SPRY^-RbBP5^330–375^ (PDB ID: 5F6L)^16^ into the cryo-EM maps (Fig. 3a, b). The secondary structural components of MLL1^SET^,^33^ including the α-helices and β-hairpin of the SET-I, SET-N, and SET-C domains (dotted circles), fitted well into the cryo-EM map (Fig. 3b). Similar to MLL1^SET^, distinctive features of WDR5 and ASH2L^SPRY^ were also well-defined in the cryo-EM structure (Fig. 3a, b). Importantly, the WDR5-MLL1^SET^-ASH2L^SPRY^ subcomplex did not make direct contacts with nucleosomal DNA, which was experimentally confirmed by the gel mobility assays (Supplementary Fig. 5d). The catalytic site of the MLL1^SET^ domain was outwardly orientated, conferring distance restraint on substrate accessibility (see below). In comparison with the NCP-free yeast SET1 complexes^20,21^, no major conformational change was detected in MLL1^RWSAD^ upon NCP binding (Fig. 3c).

### Dynamic ASH2L-NCP interaction is critical for H3K4me3

**Fig. 4:**
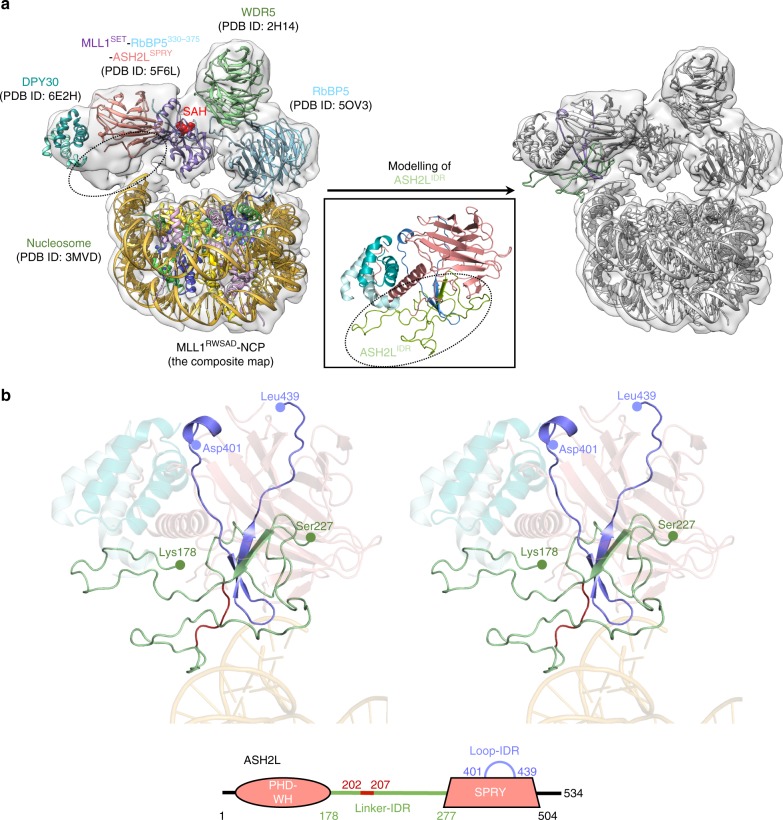
ASH2L interacts with the nucleosomal DNA through IDRs. **a** Structure prediction of ASH2L^IDR^. The structure of ASH2L IDR regions was not available and thus not assigned in the corresponding cryo-EM map (dashed circle). The structure prediction approach was employed to model ASH2L IDR regions as described in the STAR methods. Linker-IDR colored green and Loop-IDR colored blue in the ASH2L^IDR^ model structure. **b** Stereo-view of the ASH2L-DPY30 model structure and its contacts with DNA. The structure of ASH2L is a composite from crystal structure of ASH2L^SPRY^ (PDB ID: 5F6L)^16^ and the modeled ASH2L^IDR^. The schematics of ASH2L was shown at the bottom and key residues 202–207 in ASH2L^IDR^ were highlighted in red.

The second major interaction between the MLL1 core complex and the NCP was mediated by the intrinsically disordered regions (IDRs) of ASH2L (Figs. 1d, 3c). The ASH2L-NCP interface was found to be highly dynamic in solution (Supplementary Fig. 3e and Supplementary Movie 1), thereby rendering it difficult to visualize the molecular details. Similar dynamic behaviour was observed for the IDR of the yeast homologue Bre2/Cps60 (Fig. 3c), which was not resolved in the cryo-EM structure of the yeast SET1 complex^21^. Given that the crystal structure of the full-length human ASH2L has yet to be reported, we employed the protein structure prediction approach using the iterative template-based fragment assembly refinement (I-TASSER) method^34,35^. The crystal structure of yeast Bre2 was used as template (PDB ID: 6CHG)^20^ to build the ASH2L plant homeodomain-wing helix (PHD-WH)/IDRs model (Fig. 4a and Supplementary Fig. 6a). After resolving minor clashes, we were able to reliably dock ASH2L IDRs into the cryo-EM map of MLL1^RWSAD^-NCP (Fig. 4a and Supplementary Fig. 6a, b). The MLL1^RWSAD^-NCP model revealed that ASH2L IDRs interacted with the SHL7 of nucleosomal DNA (Figs. 1d and 4b). Surprisingly, the PHD-WH domain of ASH2L was located outside the region encompassed by the cryo-EM map, despite its reported function in DNA binding (Supplementary Fig. 7a)^36,37^.

**Fig. 5:**
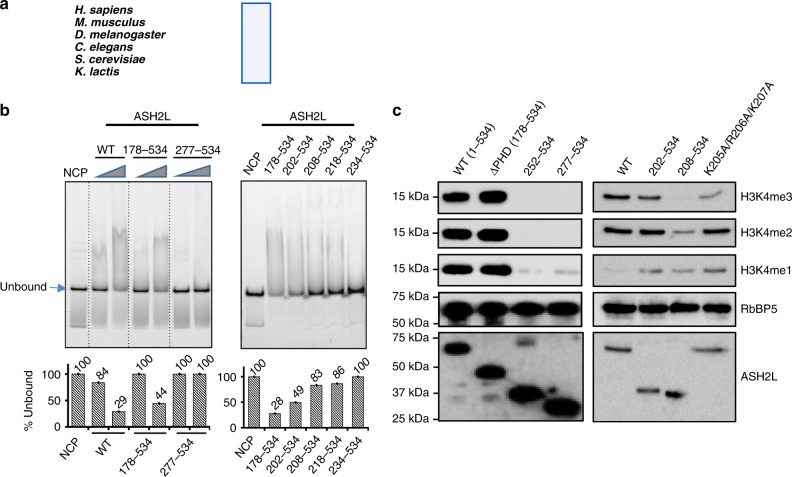
ASH2L Linker-IDR is important for NCP binding and methyltransferase activity. **a** Multiple sequence alignment of ASH2L Linker-IDR region (residues 202–254). The blue box indicated _205_-KRK-_207_, key residues for NCP recognition. **b** Top, electrophoretic mobility shift assay of ASH2L and ASH2L mutants as indicated on top. Bottom, the unbound NCP in the gel image was quantified by ImageJ and presented after normalization against the NCP alone signal, which was arbitrarily set as 1 (100%). This experiment was repeated separately to confirm the main conclusions. **c** Immunoblot to detect in vitro histone methyltransferase activity with the NCP as the substrate. Reconstituted MLL1^RWSAD^ complexes containing wild-type and mutant ASH2L, used as indicated on top. Immunoblots of RbBP5 and ASH2L included as controls.

Our MLL1^RWSAD^-NCP model pinpointed a short stretch of positively charged residues (i.e., K205/R206/K207) in the ASH2L Linker-IDR with potential to make contacts with nucleosomal DNA (Fig. 4b). These positively charged residues were found to be highly conserved in the ASH2L homologs of higher eukaryotes (Fig. 5a). To biochemically validate the structure model, we first confirmed that ASH2L directly interacted with the NCP, resulting in a mobility shift in the native gel (Fig. 5b and data not shown). Deletion of both PHD-WH (residues 1–178) and Linker-IDR (residues 178–277), but not PHD-WH alone, abolished ASH2L interaction with the NCP (Fig. 5b). Further truncation of the ASH2L Linker-IDR enabled us to establish that residues 202–207 were important for NCP interaction, consistent with the structure model (Fig. 4b). Binding of ASH2L to the NCP was shown to be critical for MLL1 activity on the NCP. Deletion of ASH2L Linker-IDR completely abolished the MLL1 activity on the NCP (Fig. 5c, left). Similarly, deletion of ASH2L residues 202–207 or mutations of residues K205/R206/K207 to alanine also significantly reduced MLL1 H3K4me3 activity on the NCP (Fig. 5c, right), but not on free H3 (Supplementary Fig. 7b). These results, together with those for RbBP5, indicate that MLL1-NCP interactions specifically promote the tri-methylation of H3K4. Notably, deletion of ASH2L Linker-IDR led to a more pronounced reduction in overall H3K4me, thereby suggesting that Linker-IDRs may contribute to MLL1 regulation through additional uncharacterized mechanisms (see Discussion).

### Alignment of MLL1^SET^ at the nucleosome dyad

**Fig. 6:**
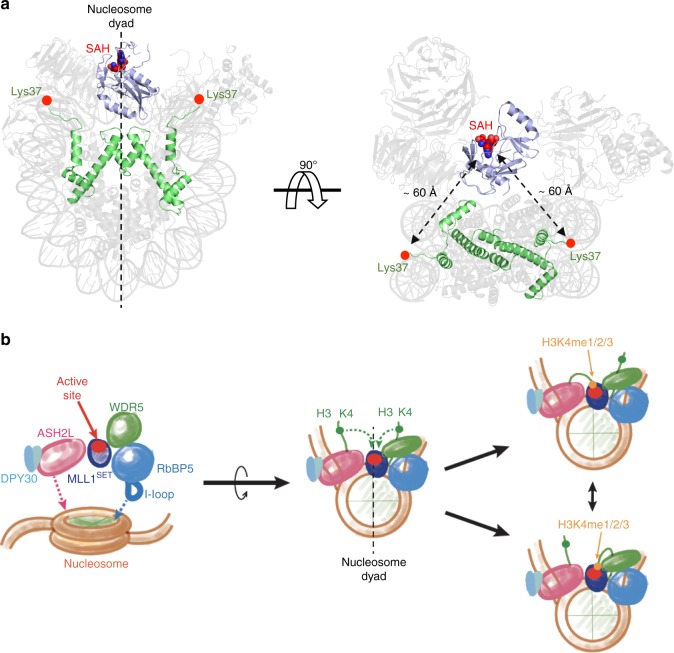
The MLL1^SET^ domain aligns at the nucleosome dyad. **a** MLL1^SET^ (slate) and two copies of histone H3 (green) were highlighted against the faded MLL1^RWSAD^-NCP structure. The SAH molecule represented as a sphere and marked the catalytic active site of MLL1^SET^. **b** Schematic model of NCP recognition mediated by the MLL1 core complex. The I-loop of RbBP5 and the active site of MLL1^SET^ were colored in blue and red, respectively.

Given the binding of RbBP5 and ASH2L at the edge of the NCP (SHL1.5 and SHL7), the catalytic MLL1^SET^ domain was positioned at the nucleosome dyad with the active site of the MLL1^SET^ domain pointing outward (Fig. 6a). Both histone H3 tails emanated from between two gyres of nucleosomal DNA, with Lys37 as the first observable residue on histone H3 N-terminal tails (Fig. 6a). The distance between Lys37 on each H3 tail and the active site of MLL1^SET^ was ~60 Å, allowing active-site access to H3 K4 and K9, but not to H3K27 (Fig. 6a). More importantly, the active site near nucleosome dyad had almost equal accessibility to K4 residues on both H3 tails (Fig. 6a). As MLL1^SET^ is a non-processive enzyme^19^, physical tethering and symmetric accessibility of the MLL1^SET^ domain to both H3K4 substrates in the NCP likely play significant roles in promoting its activity towards higher H3K4me states on the NCP (Fig. 6b and see Discussion).

## Discussion

Our cryo-EM structure reveals a unique mode of the NCP interaction by chromatin-associating complexes. Previous structural studies highlighted the importance of acidic patch, which is a negatively charged and solvent exposed surface in histones H2A and H2B^30^, in NCP–protein interactions. The acidic patch is recognized by the NCP-interacting proteins in many cases through diverse arginine-finger motifs (e.g., LANA^2^, RCC1^3^, and 53BP1^38^). In contrast, binding of the MLL1 core complex to the NCP does not involve the acidic patch. Instead, the main contributors are the electrostatic interactions between positively charged residues in RbBP5 and ASH2L and the DNA backbone in the NCP. Extensive DNA interactions were also observed for the histone H3K27 methyltransferase PRC2 in complex with dinucleosomes^39^. However, unlike PRC2, all MLL1-NCP interactions occur within a single nucleosome. It is possible that additional interactions between the MLL1 complex and chromatin are necessary to engage adjacent nucleosomes and spreading the H3K4me marks on chromatin^24^.

Our structure shows that the I-loop of RbBP5 inserts between the H4 tail and nucleosomal DNA (SHL1.5) and this interaction specifies the orientation of MLL1 complex on the NCP. Having docked via RbBP5, the distance between RbBP5 and ASH2L (~70 Å) limits ASH2L binding on the NCP to SHL7 (Supplementary Fig. 7c). Although RbBP5 did not contribute significantly to the NCP binding (Fig. 2g), dual recognition through both specific and nonspecific interactions of RbBP5 and ASH2L enables a unique configuration of the MLL1 core complex on the NCP for optimal catalytic activity for H3K4me3 (Fig. 6b). Notably, H4 interactions are also used by other chromatin-interacting proteins, e.g., DOT1L, SNF2h, and ISWI^31,32,40–44^, raising the possibility that H4 tail is another general protein docking site on the NCP in addition to the acidic patch.

The findings of the present study indicate that human MLL1 core complex and the yeast SET1 complexes have similar overall architecture^20,21^, with the catalytic MLL1^SET^ domain sandwiched by RbBP5-WDR5 (Swd1-Swd3 in *Kluveromyces lactis*/Cps50-Cps30 in *Saccharomyces cerevisiae*) and ASH2L-DPY30 (Bre2-Sdc1/Cps60-Cps25 in yeast) on each side (Fig. 3c). Furthermore, the NCP-free the yeast SET1 complex^20^ overlays well with MLL1^RWSAD^ on the NCP. Despite overall conservation, the interfaces of RbBP5 and ASH2L with the NCP are not well conserved in homologous yeast Swd1/Cps50 and Bre2/Cps60 proteins (Fig. 5a and Supplementary Fig. 5b). In particular, we found that the I-loop in the yeast Swd1/Cps50 protein is considerably shorter than that in human MLL proteins, and the A-loop is absent (Supplementary Fig. 5a), suggesting potential divergence of detailed yeast SET1-NCP interactions at the molecular level. In contrast, sequence alignments show significant conservation of the I-/A-loops as well as the basic residues in RbBP5 and ASH2L homologs, respectively, in higher eukaryotes. It supports a highly conserved mechanism by which the MLL family enzymes engage chromatin in higher eukaryotes. It also implies the functional importance of these regions in H3K4me regulation. Consistently, recent genome sequencing studies have identified mutations in the conserved regions of ASH2L and RbBP5 in human malignancies^45,46^, which warrants future studies.

Although our paper is under consideration, cryo-EM structures of MLL1-NCP, MLL1-ubiquitinated NCP (ubNCP), and MLL3-ubNCP are reported^47^. Comparison of our structure with the published MLL1-NCP structures revealed that our structure is similar to one of the reported MLL1-NCP models (mode 2, EMD-0695 and PDB ID: 6KIZ). Notwithstanding overall similarity, major differences are found in the ASH2L IDR and DPY30 regions. Specifically, despite highly dynamic features (Supplementary Fig. 3e), the density of ASH2L IDR and DPY30 is more visible in our cryo-EM map. This could be due to different emphasis on global high-resolution^47^ versus visualization of ASH2L IDR and DPY30 at the expense of resolution, which lead to selection of different particle population for refinement. In our structure, we were able to visualize the ASH2L-NCP contacts that serve as basis for modeling and biochemical validation. Our study highlights significance of ASH2L IDR in NCP recognition and its contribution to H3K4 tri-methylation. Notably, Huang and colleagues have shown that the MLL1-NCP is able to adopt another conformation on the NCP (mode 1, EMD-0694 and PDB ID: 6KIX). In this mode, Quad-R and I-loop of RbBP5 do not interact with the NCP in the same manner as we have shown in our study. Only two of the Quad-R residues are involved in nucleosomal DNA recognition and the I-loop, which does not contain any positively charged Arg or Lys residue, is positioned close to nucleosomal DNA. As our biochemical assay has shown that both Quad-R and I-loop dramatically affect H3K4 tri-methylation activity. We speculate whether our structure represents an active conformation for optimized H3K4 tri-methylation. It is important to examine whether deletion or mutations of key residues identified in second MLL1-NCP mode specifically affects H3K4me3. Interestingly, mode 1 of MLL1-NCP interaction is more prevalent in the MLL1-ubNCP complex, suggesting that H2B-ub is able to further stabilize MLL1 interaction with the histone core. This stable anchoring may underlie the modest increase of MLL1 activity on ubNCP.

Previous single turnover kinetic experiments have revealed that the MLL1 core complex deploys a non-processive mechanism for catalysis^19^, requiring capture and release of H3K4 after each round of methylation. In the present study, we demonstrate that the MLL1 core complex stably associates with NCP via RbBP5 and ASH2L and uniquely positions the MLL1^SET^ domain at the nucleosome dyad with near symmetric access to both H3K4 substrates (Fig. 6a). Stable settlement on the NCP allows close physical proximity and optimal orientation of the MLL1^SET^ catalytic site to both H3K4 substrates on the NCP, which significantly favors the kinetics of successive methylation reactions. We demonstrated that disruption of the MLL1-NCP interactions significantly reduces H3K4me3 activity on the NCP without affecting methylation activity on free H3. The positioning of the MLL1^SET^ domain at the nucleosome dyad also raises the possibility of potential interplay with linker histones, which bind near the nucleosome dyad^48–50^. It would be interesting to examine whether linker histone inhibits MLL1 activity and thus indirectly promotes repressive chromatin environment in future.

## Methods

### Protein expression and purification

The core subunits of the MLL1^RWSAD^ complex (MLL1^SET, 3762–3969^, ASH2L1–534 and mutants, RbBP51–538 and mutants, WDR523–334, and DPY301–99) were expressed and purified using the pET28a His_6_-small ubiquitin-related modifier vector as previously described^51^. Briefly, individual protein constructs were transformed and grown by traditional heat-shock methods. Main cultures were inoculated, grown to 0.6–0.8 OD_600_, and cooled to 20 °C. After shaking at 20 °C for 45 min, protein expression was induced with 0.4 mM isopropyl β- d-1-thiogalactopyranoside (IPTG) for 16–18 h. Individual components of the MLL1^RWSAD^ complex were purified on Ni-NTA column and equimolar quantities were mixed, concentrated, and purified by gel filtration chromatography.

Mutations of RbBP5 and ASH2L were generated by overlapping PCR-based mutagenesis. Full-length *Xenopus laevis* histones H2A, H2B, H3, and H4 were expressed and purified using the one-pot purification method^52^. Briefly, histone constructs were transformed as described above. Histone expression was induced using 0.4 mM IPTG and were grown for 3 h (H2A, H2B, and H3) or 2 h (H4) at 37 °C. Histones were then purified by first combining equimolar amounts, sonicating, centrifugation, and then isolated from inclusion bodies using denaturing conditions (8 M guanidinium HCl, 20 mM sodium acetate (pH 5.2), 10 mM dithiothreitol (DTT)). Octamer refolding was conducted by dialyzing into high-salt buffer (20 mM Tris-HCl (pH 8.0), 2 M NaCl, 2 mM β-mercaptoethanol) overnight. Supernatant was purified over Ni-NTA, pooled, concentrated, and purified over gel filtration. Octamer fractions were combined and concentrated to prepare for nucleosome reconstitution. Assembly of the NCP using 147 base pair Widom 601 DNA was done by salt dialysis. Briefly, concentrated fractions were dialyzed into high-salt buffer (20 mM Tris-HCl (pH 7.5), 2  M NaCl, 1 mM EDTA, 1 mM DTT) and concentration was measured by UV at 280 nm. One hundred and forty-seven base-pair Widom DNA and octamer were combined in 1:1 molar ratio in high-salt buffer and overnight linear salt gradient was done using peristaltic pump adding in low-salt buffer (20 mM Tris-HCl (pH 7.5), 0.2 M NaCl, 1 mM EDTA, 1 mM DTT) at 1 mL/min at 4 °C. Final nucleosome construct was then dialyzed into 20 mM cacodylate (pH 6.0), 1 mM EDTA for storage at 4 °C.

### In vitro histone methyltransferase assay

The in vitro histone methyltransferase assay was carried out by incubating the MLL1^RWSAD^ complex (0.3 µM) with either nucleosome (0.965 µM) or free recombinant histone H3 (0.098 µM) for 1 h at room temperature. The reaction buffer contained 20 mM Tris-HCl, pH 8.0, 50 mM NaCl, 1 mM DTT, 5 mM MgCl_2_, and 10% v/v glycerol in a total volume of 20 µL. Reactions were quenched with 20 µL of 2× Laemmli Sample Buffer (Bio-Rad catalog number #161–0737). H3K4 methylation was detected by western blotting using antibodies for H3K4me1 (1:20,000, Abcam catalog number ab8895), H3K4me2 (1:40,000, EMD-Millipore catalog number #07–030), or H3K4me3 (1:10,000, EMD-Millipore catalog number #07–473) for either 1 h at room temperature or overnight at 4 °C. The blot was then incubated with IgG-HRP (Santa Cruz Biotechnology catalog number #sc-486) for 1 h at room temperature. The membrane was developed using ECL (Pierce catalog number #32106) and visualized by chemiluminescence (Bio-Rad ChemiDoc Imaging System).

### Electrophoretic mobility shift assay

EMSA assay was carried out using 0.1 µM nucleosomes and increasing concentration of MLL1 subunits. The protein mixture was run on the 6% 0.2× TBE gel that was pre-run for 1.5 h, 150 V at 4 °C. The gel was visualized by incubating in 100 mL of TAE with 1:20,000 diluted ethidium bromide for 10 min at room temperature. Gels were then incubated in distilled water for 10 min and visualized by UV transillumination (Bio-Rad ChemiDoc Imaging System). The results were quantified by ImageJ software.

### His_6_ pull-down assay

His_6_-fusion proteins were incubated with the NCP in BC150 (20 mM Tris-HCl, pH 7.5, 350 mM NaCl, 20 mM imidazole, 0.05% v/v NP-40, 10 mM DTT, 1 mg/ml bovine serum albumin, phenylmethylsulfonyl fluoride, and inhibitor cocktail) for 2 h at 4 °C. After several washes with BC150, the beads were boiled in SDS loading buffer and analyzed by western blotting.

### Cryo-EM sample preparation

The cryo-EM sample was prepared by the GraFix method^53^. Specifically, the reconstituted MLL1^RWSAD^ complex (30 μM) was incubated with nucleosomes (10 μM) in GraFix buffer (50 mM HEPES, pH 7.5, 50 mM NaCl, 1 mM MgCl_2_, 1 mM TCEP) with added 0.5 mM SAH for 30 min at 4 °C. The sample was applied onto the top of the gradient solution (0–60% glycerol gradient with 0–0.2% glutaraldehyde, in GraFix buffer) and was centrifuged at 48,000 r.p.m. at 4 °C for 3 h. After ultracentrifugation, 20 μl fractions were manually collected from the top of the gradient. The crosslinking reaction was terminated by adding 2 μl of 1 M Tris-HCl, pH 7.5 into each fraction. Glycerol was removed by buffer exchange via concentration and centrifugation in GraFix buffer using centrifugal concentrator (Sartorius Vivaspin 500) before making cryo-EM grids.

### Cryo-EM data collection and processing

A protein sample at 1 mg/ml concentration was plunge-frozen on 200 mesh quantifoil R1.2/1.3 grids (Electron Microscopy Sciences) using a Mark IV Vitrobot (Thermo Fisher Scientific) with settings as 4 °C, 100% humidity, and 4 s blotting time. Cryo-EM grids were imaged on a FEI Titan Krios operating at 300 KV at liquid nitrogen temperature. The Gatan K2 Summit direct electron detector was used at a nominal magnification of 29,000× in a counting mode with a pixel size of 1.01 Å/pixel. A dose rate of 8 electrons/Å^2^/s and defocus values ranging from −1.5 to −3.5 μm were used. Total exposure of 8 s per image was dose-fractionated into 40 movie frames, resulting in an accumulated dose of 64 electrons per Å^2^. A total of 4717 movies were collected for the MLL1^RWSAD^-NCP dataset.

Micrograph movie stacks were first subjected to MotionCor2 for whole-frame and local drift correction^54^. For each micrograph, CTFFIND4.1 was used to fit the contrast transfer function^55^. The estimated resolution of micrographs lower than 5 Å were excluded from further processing, which resulted in 3896 micrographs. Particle picking was performed using the Warp^56^, which picked total 712,198 particles. Using particle coordinates obtained from the Warp, the particles were extracted with the box size of 350 Å using RELION 3 program package^57^. Extracted particles were then imported into cryoSPARC for 2D classification in 200 classes. After removal of bad classes, the total of 694,180 particles were subjected to ab initio three-dimensional (3D) classification (Supplementary Fig. 2). The major class (323,408 particles) contained the MLL1 core complex and the NCP, which was then subjected for the heterogeneous refinement. This led to the identification of ten subclasses. One subclass showed the partial cryo-EM density for the MLL1 core complex, thus excluded for the further processing. The remaining nine subclasses (252,109 particles) maintained intact MLL1^RWSAD^-NCP complex. These nine subclasses also used for the rigid-body fitting of individual component of the MLL1^RWSAD^-NCP complex to visualize the dynamics of each component against the NCP (Supplementary Fig. 3e and Supplementary Movie 1). Particles (252,109) were imported in RELION and performed the 3D classification without alignment (10 classes, 35 cycles, *T* = 40, binary mask: 8 pixels/soft mask: 5 pixels). One out of 10 classes (8433 particles) exhibited, the well-defined map of MLL1^RWSAD^-NCP with clear features of WD40 repeat blades in RbBP5 and WDR5, as well as densities of MLL1^SET^ and ASH2L-DPY30 compared with others (Supplementary Fig. 2). These particles were used for 3D refinement in RELION and post-processed to a resolution of 6.2 Å and a B factor of −189 Å^2^. This cryo-EM map was local filtered using RELION to the local resolution to avoid over-interpretation (Fig. 1c).

To obtain a cryo-EM map for RbBP5-NCP and MLL1^RWS^-NCP subcomplexes, we utilized RELION’s multibody refinement procedure with 252,109 particles (Supplementary Fig. S2). RbBP5-NCP (32,563 particles), MLL1^WSAD^, MLL1^RWS^-NCP (21,114 particles), and MLL1^AD^ were separately masked during the multibody refinement^58^. After multibody refinement, we generated the partial signal subtracted particle sets for RbBP5-NCP and MLL1^RWS^-NCP using relion_flex_analysis^57^. Further 3D classifications without alignment (5 classes, 35 cycles, *T* = 40, and binary mask: 5 pixels/soft mask: 5 pixels for RbBP5-NCP; 10 classes, 35 cycles, *T* = 40, and binary mask: 5 pixels/soft mask: 5 pixels for MLL1^RWS^-NCP) were performed and the best maps based on the resolution and occupancy of RbBP5 and MLL1^RWS^ densities were selected for further refinement and post-processing (Supplementary Fig. 2). The reported final resolution of each cryo-EM structure was estimated by RELION with Fourier shell correlation (FSC) at criteria of 0.143 (Supplementary Fig. 3a-c).

### Modeling, rigid-body fitting, and model refinement

We built a 3D atomic model of the human ASH2L protein by I-TASSER^34,35^ assisted by deep-learning based contact-map. The fragment-guided molecular dynamics refinement software, FG-MD^59^, was utilized to remove the steric clash between ASH2L model and other molecules and further refine the local structures (Supplementary Fig. 6b). Finally, our in-house EM-fitting software, EM-Ref (Zhang et al, in preparation), was used to fit the ASH2L model and other parts of human MLL1 core complex to the density maps to get final atomic models.

I-TASSER utilized LOMETS, which consisted of 16 individual threading programs^60^, to generate templates as the initial conformation. Human ASH2L protein consisted of three domains, whereas the 2nd, 3rd domains (Linker-IDR and ASH2L^SPRY^), and C-terminal SDI motif can be covered by templates (PDB ID: 6E2H and 6CHG, B-chain, crystal structure of the yeast SET1 H3K4 methyltransferase catalytic module^20^) in most of the top threading alignments. The 1st domain (PHD-WH domain) was covered by another template (PDB ID: 3S32, A chain, the crystal structure of ASH2L N-terminal domain)^37^. Therefore, these three proteins were used as the main templates for building the full-length ASH2L model, where structural assembly simulation was guided by the contact-maps from the deep-learning program, ResPRE^61^. Finally, the first model of I-TASSER was selected as the potential ASH2L model, where the estimated TM-score^62^ for the C-terminal domain is 0.71 ± 0.12, suggesting that the confidence of the I-TASSER model is high. Superposing ASH2L model (Linker-IDR and ASH2L^SPRY^) with the experimental structure (ASH2L^SPRY^) is shown in Fig. 4a.

Monte Carlo simulation was employed to fit and refine the complex model structures based on the experimental density map. During the MC simulations, individual domain structures were kept as the rigid body, where global translation and rotation of the domains were performed, which would be accepted or rejected based on Metropolis algorithm^63^. The total number of translation and rotation was 50,000 in the MC simulation. The MC energy function used in the simulation was a linear combination of correlation coefficient (CC) between structural models and the density map data and the steric clashes between the atomic structures, i.e.,

1 $$E_{main} = \,\, w_1\left( {1.0 - \frac{{\mathop {\sum }\nolimits_{y \in DM} \left( {\rho _0\left( y \right) - \overline {\rho _0} } \right)\left( {\rho _c\left( y \right) - \overline {\rho _c} } \right)}}{{\sqrt {\mathop {\sum }\nolimits_{y \in DM} \left( {\rho _0\left( y \right) - \overline {\rho _0} } \right)^2} \sqrt {\mathop {\sum }\nolimits_{y \in DM} \left( {\rho _c\left( y \right) - \overline {\rho _c} } \right)^2} }}} \right)\; \\ + \, w_2\mathop {\sum}\limits_{i \in L} {\mathop {\sum}\limits_{j \in L,i \ne j} {\varepsilon _{ij}\left[ {\left( {\frac{{r_{ij}}}{{d_{ij}}}} \right)^{12} - 2\left( {\frac{{r_{ij}}}{{d_{ij}}}} \right)^6} \right]} }$$

where *ρ*_c_(*y*) was the calculated density map on grid^64^. *ρ*_o_(*y*) was obtained from the experimental density map. $\bar \rho _c$ and $\bar \rho _o$were the average of calculated density map and experimental density map, respectively. DM and *L* represented the density map and the length of protein, respectively. *d*_*ij*_ was the distance between the two atoms *i* and *j*. *r*_*ij*_ was the sum of their van der Waals atomic radii and *ε*_*ij*_ was the combined well-depth parameter for atoms *i* and *j*, which were all taken from the CHARMM force field^65^. *w*_1_ = 100 and *w*_2_ = 1 were the weights for CC item and clash item, respectively.

For the nucleosome model, the crystal structure of nucleosome (PDB ID:3MVD)3 was used for rigid-body fitting. In the cryo-EM structure of RbBP5-NCP, the histone H4 tail region was manually rebuilt where the density allowed using the program COOT. Three model structures of MLL1^RWSAD^-NCP, RbBP5-NCP, and MLL1^RWS^-NCP were subjected to the real-space refinement using PHENIX^66^ after rigid-body fitting using Chimera^67^. Crystal structures of mouse RbBP5 (PDB ID: 5OV3)^27^, human WDR5 (PDB ID: 2H14)^28^, human MLL1^SET^-ASH2L^SPRY^-RbBP5^330–375^ (PDB ID: 5F6L)^16^, a DPY30 dimer (PDB ID: 6E2H)^29^, the 601-NCP (PDB ID: 3MVD)^3^, and the ASH2L IDR model structure from I-TASSER^34,35^ were used for the rigid-body fitting and following real-space refinement. Validations of three model structures were performed by MolProbity^68^. The final structures were further validated by calculating map-model FSC curves using phenix.mtriage in the PHENIX program package (Supplementary Fig. 3d). The computed FSC between the model and map agreed reasonably well as shown in Supplementary Fig. 3d. Statistics for data collection, refinement, and validation summarized in Supplementary Table 1.

### Model quality estimation of the ASH2L IDR region

The estimated TM-score of the entire model is 0.67 ± 0.13 and the CC between the predicted model and cryo-EM density map was 0.696. These data showed that the predicted model was a confident model and there was a good fitting between the predicted model and the density map. To further check the local model quality, especially for the IDR region, we gave the residue-level B-factor predicted by ResQ^69^ and the CC score between the predicted model and the cryo-EM density map in Supplementary Fig. 8.

B-factor was estimated by ResQ, which uses support vector regression that makes use of the local structural information between the model and (1) threading templates, (2) structure alignment templates, (3) reference decoys, and (4) sequence-based secondary structure and solvent accessibility predictions^69^. Since IDR region of the ASH2L model are mainly loops, this region was more flexible and had relatively high B-factors (Supplementary fig. 8). However, it is difficult to simply say that IDR region has a good model quality. Therefore, after fitting the model to the density map, we gave the residue-level CC score between each residue of the ASH2L IDR model and the corresponding residue of the density map (Supplementary Fig. 8) to further test the quality of the IDR region. The residue-level CC score can be calculated using Eq. (2). The masking distance in Eq. (2) is 5 Å if every atom is used to compute $\rho _c$, where $\rho _c$ is the density map calculated from the fitted model. $\rho _o$ is experimental density map. *y* is the grid point where its distance to atoms of residue *i* is < 5 Å^64^. Positive value of CC score indicates good fitting quality of the model and the density map. In the IDR region, especially for the DNA binding interface residues (residues 205–207), most of all residues had positive CC scores, indicating that by combining the information from the predicted model and the density map, our model for the IDR region is trustable.

2 $$CC(R_i) = \frac{{\mathop {\sum }\nolimits_{y \in R_i} (\rho _o(y) - \bar \rho _o)(\rho _c(y) - \bar \rho _c)}}{{\sqrt {\mathop {\sum }\nolimits_{y \in R_i} (\rho _o(y) - \bar \rho _o)^2} \sqrt {\mathop {\sum }\nolimits_{y \in R_i} (\rho _c(y) - \bar \rho _c)^2} }}$$

### Reporting summary

Further information on research design is available in the Nature Research Reporting Summary linked to this article.

## Supplementary information

Supplementary Information

Description of Additional Supplementary Files

Supplementary Movie 1

Reporting Summary

## Data Availability

The data supporting this study are available from the corresponding authors upon reasonable request. The structural data has been deposited to the PDB and EMDB. The accession numbers for the MLL1^RWSAD^-NCP, RbBP5-NCP, and MLL1^RWS^-NCP cryo-EM structures are PDB: 6PWV and EMDB: EMD-20512; PDB 6PWX and EMDB: EMD-20514; and PDB: 6PWW and EMDB: EMD-20513, respectively. The source data underlying Figs. 1b, 2c-d, 2g, and 5c, and Supplementary Figs. 1d and 5c are provided as a Source Data File.

## Source data

Source Data
