# Publisher Correction: Cryo-EM structure of the human MLL1 core complex bound to the nucleosome

**DOI:** 10.1038/s41467-020-14973-y

**Published:** 2020-02-27

**Authors:** Sang Ho Park, Alex Ayoub, Young-Tae Lee, Jing Xu, Hanseong Kim, Wei Zheng, Biao Zhang, Liang Sha, Sojin An, Yang Zhang, Michael A. Cianfrocco, Min Su, Yali Dou, Uhn-Soo Cho

**Affiliations:** 1 Department of Biological Chemistry, University of Michigan, Ann Arbor, Michigan 48109 USA; 2 Department of Pathology, University of Michigan, Ann Arbor, Michigan 48109 USA; 3 Computational Medicine and Bioinformatics, University of Michigan, Ann Arbor, Michigan 48109 USA; 4 Life Sciences Institute, University of Michigan, Ann Arbor, Michigan 48109 USA

**Keywords:** Cryoelectron microscopy, Epigenetics

Correction to: *Nature Communications* 10.1038/s41467-019-13550-2, published online 5 December 2019.

The original version of this Article contained an error in Fig. 5. In panel a, the multiple sequence alignment of ASH2L Linker-IDR region was not displayed.

This has now been corrected in both the PDF and HTML versions of the Article.

