# Supplementary material for: Cryo-EM structure of the human MLL1 core complex bound to the nucleosome
Source: Nat Commun. 2019 Dec 5;10:5540. doi: 10.1038/s41467-019-13550-2 (PMC6895043; doi:10.1038/s41467-019-13550-2)
Supplement: Supplementary file 1 — Supplementary Information [file 41467_2019_13550_MOESM1_ESM.pdf]

Supplementary Materials

Cryo-EM structure of the human MLL1 core complex bound to the nucleosome

Sang Ho Park, Alex Ayoub, Yong Tae Lee, Jing Xu, Hanseong Kim, Wei Zheng, Biao Zhang, Liang Sha, Sojin An, Yang Zhang, Michael A. Cianfrocco, Min Su, Yali Dou, and Uhn-Soo Cho

Supplementary Table 1. Cryo-EM Data Collection, Refinement, and Validation Statistics

	**MLL1^{RWSAD}-NCP (EMD-20512) (PDB:6PWV)**	**MLL1^{RWS}-NCP (EMD-20513) (PDB: 6PWW)**	**RbBP5-NCP (EMD-20514) (PDB: 6PWX)**
**Data Collection and Processing**			
Magnification	29,000		
Voltage (kV)	300		
Electron exposure (e-/Å^2)	64		
Defocus range (μm)	-1.5 to -3.5		
Pixel size (Å)	1.01		
Symmetry imposed	C1		
Initial particle images (no.)	712,198		
Final particle images (no.)	8,433	21,114	32,563
Map resolution (Å)	6.2	4.5	4.2
FSC threshold	0.143	0.143	0.143
Map resolution range (Å)	4.0-12.0	3.0-9.0	3.0-9.0
**Refinement**			
Initial model used (PDB code)	3MVD, 5OV3, 2H14, 5F6L, 6E2H	3MVD, 5OV3, 2H14, 5F6L	3MVD, 5OV3
Model resolution (Å)	6.7	4.7	4.3
FSC threshold	0.5	0.5	0.5
Map sharpening *B* factor (Å^2)	-189	-157	-100
**Model composition**			
Non-hydrogen atoms	21,781	18,174	14,411
Protein residues	2,005	1,550	1,074
Nucleotides	292	292	292
Ligands	2	2	-
***B* factor (Å^2)**			
Protein	192.99	109.71	107.65
Nucleotide	48.20	36.73	20.20
Ligand	781.00	826.18	-
**Rmsds**			
Bond lengths (Å)	0.005	0.004	0.003
Bond angles (°)	0.688	0.612	0.579
**Validation**			
MolProbity score	2.54	2.21	2.38
Clashscore	31.16	21.65	13.50
Poor rotamers (%)	1.42	1.22	3.58
**Ramachandran plot**			
Favored (%)	93.05	95.54	95.45
Allowed (%)	6.95	4.46	4.55
Disallowed (%)	0	0	0

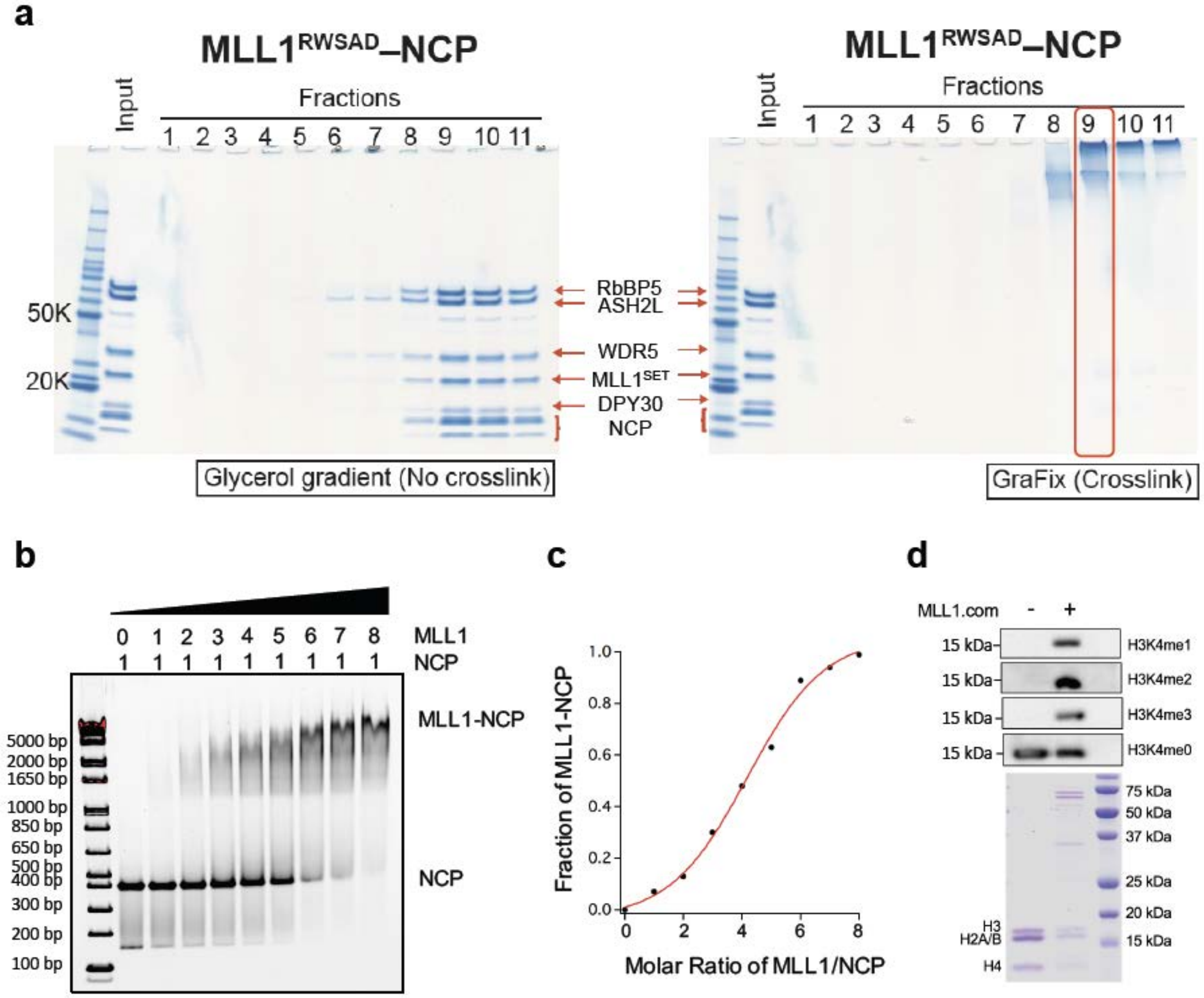

Supplementary Figure 1 | Preparation of the MLL1^{RWSAD}-NCP complex. a, MLL1^{RWSAD} complex and the NCP were incubated and isolated by glycerol-gradient (0-60%) with (right) or without (left) crosslinking. The fractions (1-11) were analyzed by SDS-PAGE. Individual components of the MLL1 core complex and the NCP were indicated. Fraction #9 from the GraFix sample (red box) was used for structural analysis. **b,** Gel mobility shift assay for the MLL1 core complex and the NCP. The molar ratio of MLL1 vs. NCP was indicated on top. **c,** Relative quantification of bound NCP (in b) by Image J was presented. The experiment was repeated twice (not shown) to confirm concentration dependent binding of MLL1 to the NCP. **d**. *In vitro* histone methyltransferase assay (HMT) for the NCP incubated with or without the MLL1 complex. No signal was detected in the immunoblot for the H3K4me1/2/3 antibodies, which confirmed their respective specificity for the modified H3 in the NCP. The antibody against unmodified H3K4 was used as the control. Coomassie gel on bottom showed components in each reaction.

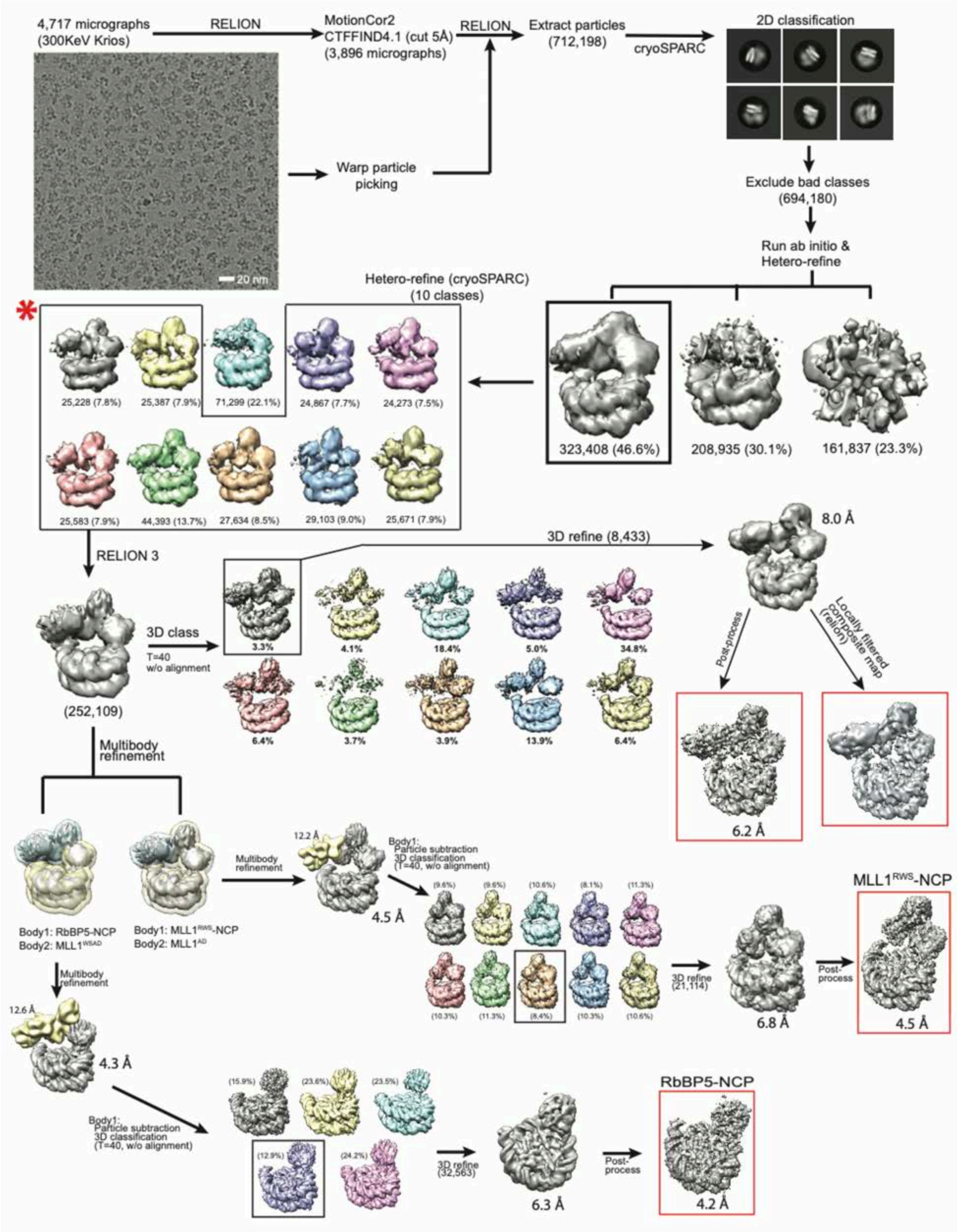

Supplementary Figure 2 | Cryo-EM data processing for the MLL1^{RWSAD}-NCP. Representative micrograph image (Titan Krios 300 KeV) and 2D classifications of the MLL1^{RWSAD}-NCP complex were shown. The particle numbers for each classification step as well

as the estimated resolution of overall and selected subcomplexes (red box) were shown at the bottom. The detailed data processing procedures of MLL1^{RWSAD}-NCP, MLL1^{RWS}-NCP, and RbBP5-NCP were described in the Methods.

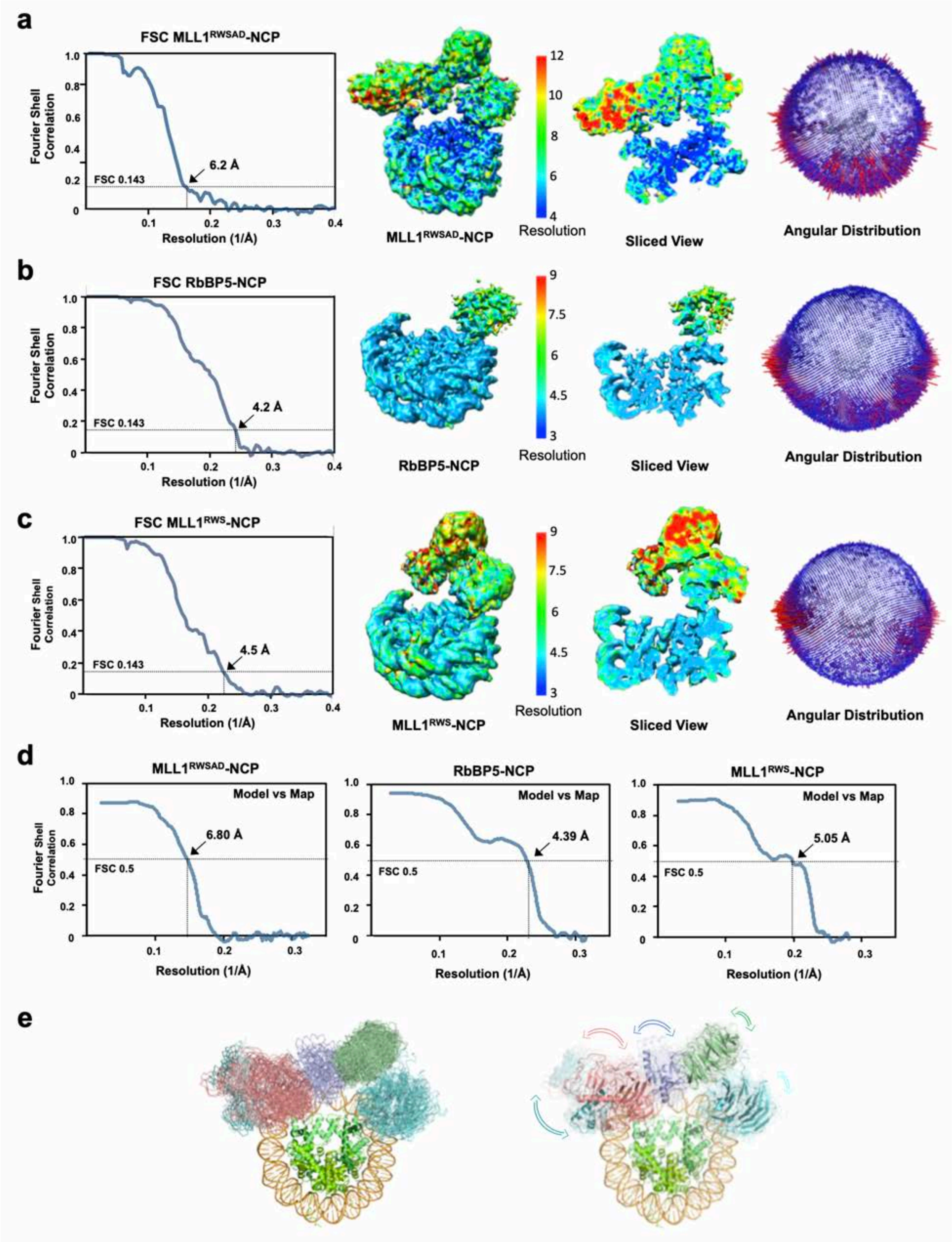

Supplementary Figure 3 | Cryo-EM map validation of MLL1^{RWSAD}-NCP, RbBP5-NCP, and MLL1^{RWS}-NCP. a-c, Fourier Shell Correlation (FSC) curves for MLL1^{RWSAD}-NCP (**a**), RbBP5-NCP (**b**), and MLL1^{RWS}-NCP (**c**) were shown on left and the corresponding local

resolution assessments by RESMAP[1] were shown on middle. The final resolution was determined using FSC=0.143 criterion, which was shown by arrowhead on the FSC curve. Angular distribution plots were shown on right. **d,** Model-map FSC curves for MLL1^{RWSAD}-NCP, RbBP5-NCP, and MLL1^{RWS}-NCP calculated by phenix.mtriage[2]. The resolution was indicated using FSC=0.5 criterion, which was shown by arrowhead on the FSC curve. **e,** Rigid body fitting of the MLL1^{RWSAD} domains into 9 cryo-EM maps of MLL1^{RWSAD}-NCP, which were shown as hetero-refine subclasses in Supplementary Fig. 2 (highlighted by *). Left, nine coordinates of the MLL1^{RWSAD} core complex were overlaid and displayed. Right, the degree of relative movement of each MLL1^{RWSAD} domain within nine coordinates was indicated by arrows. The length and orientation of each arrow indicated the degree of dynamics and moving direction of each domain.

a

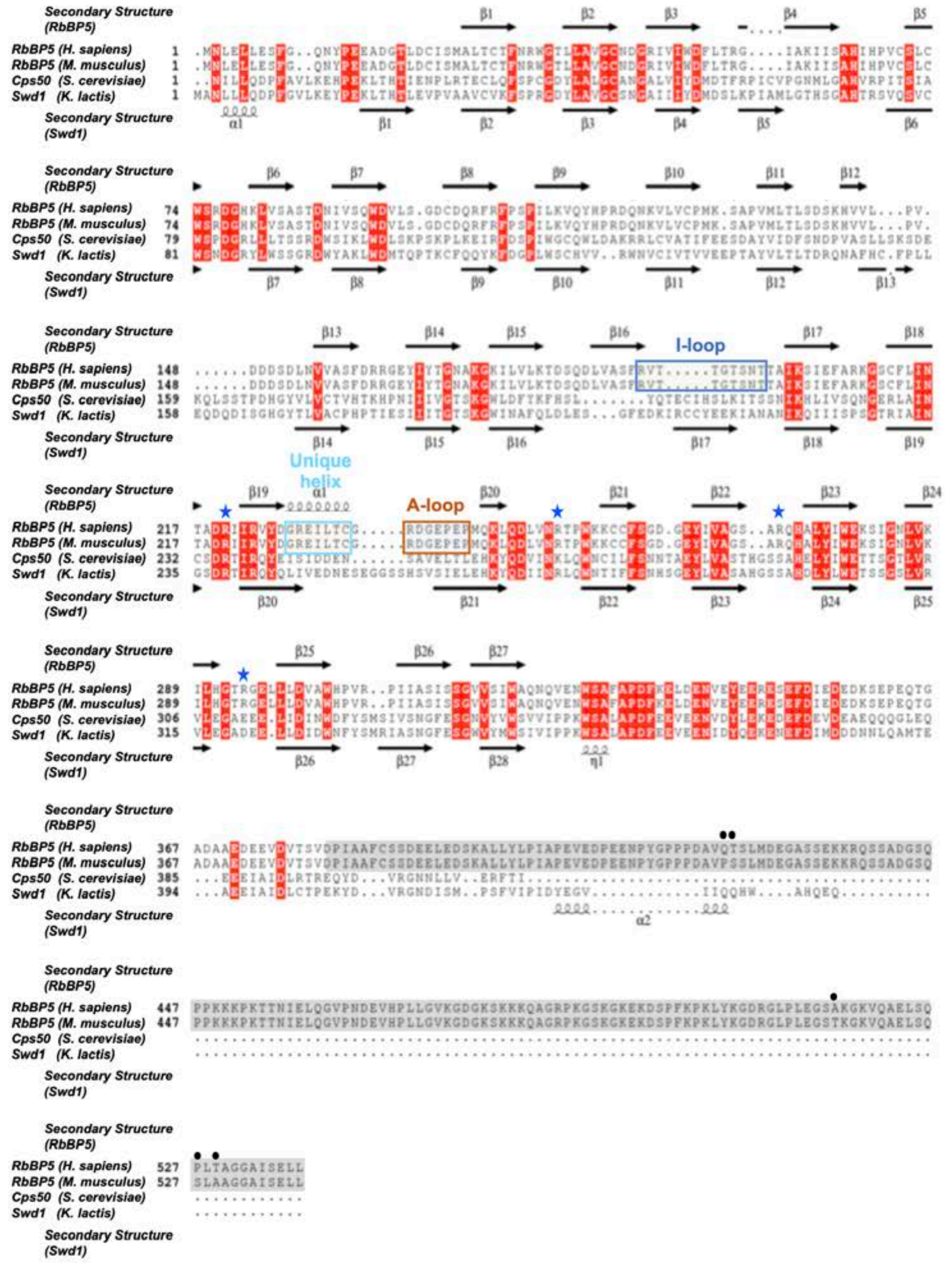

Secondary Structure (RbBP5)
RbBP5 (H. sapiens)
RbBP5 (M. musculus)
Cps50 (S. cerevisiae)
Swd1 (K. lactis)
Secondary Structure (Swd1)
I-loop
Unique helix
A-loop

b

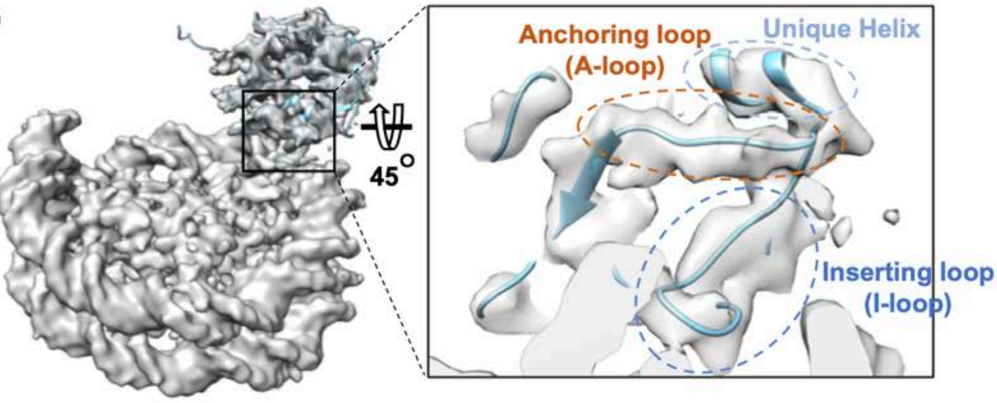

45°
Anchoring loop
(A-loop)
Unique Helix
Inserting loop
(I-loop)

Supplementary Figure 4 | The cryo-EM structure of the RbBP5-NCP subcomplex. a, The primary sequences of RbBP5 in *H. sapiens* and *M. musculus* (mammalian cells) as well as yeast homologous Swd1/Cps50 in *S. cerevisiae* and *K. lactis* were used for multiple sequence alignment. The secondary structures of mouse RbBP5 and Swd1 based on determined crystal structures were indicated on the top and bottom of the alignment, respectively. The I- and A-loops as well as the unique helix in mammalian RbBP5 were highlighted in blue, cyan, and orange boxes, respectively. Quad-R residues were shown as blue stars. Human and mouse RbBP5 had sequence divergence at five residues at C-terminus, which were indicated by black dots. The structural part of mouse RbBP5 WD40 repeats, which covers residues 1–380, are identical between human and mouse (100% sequence identity). RbBP5 C-terminus is not included in the crystal structure (grey box). **b,** Rigid-body fitting of RbBP5 (PDB ID: 5OV3) in the RbBP5-NCP cryo-EM map. Characteristic I- and A-loops as well as the unique helix (zoomed in left) of RbBP5 were fitted nicely into the cryo-EM map.

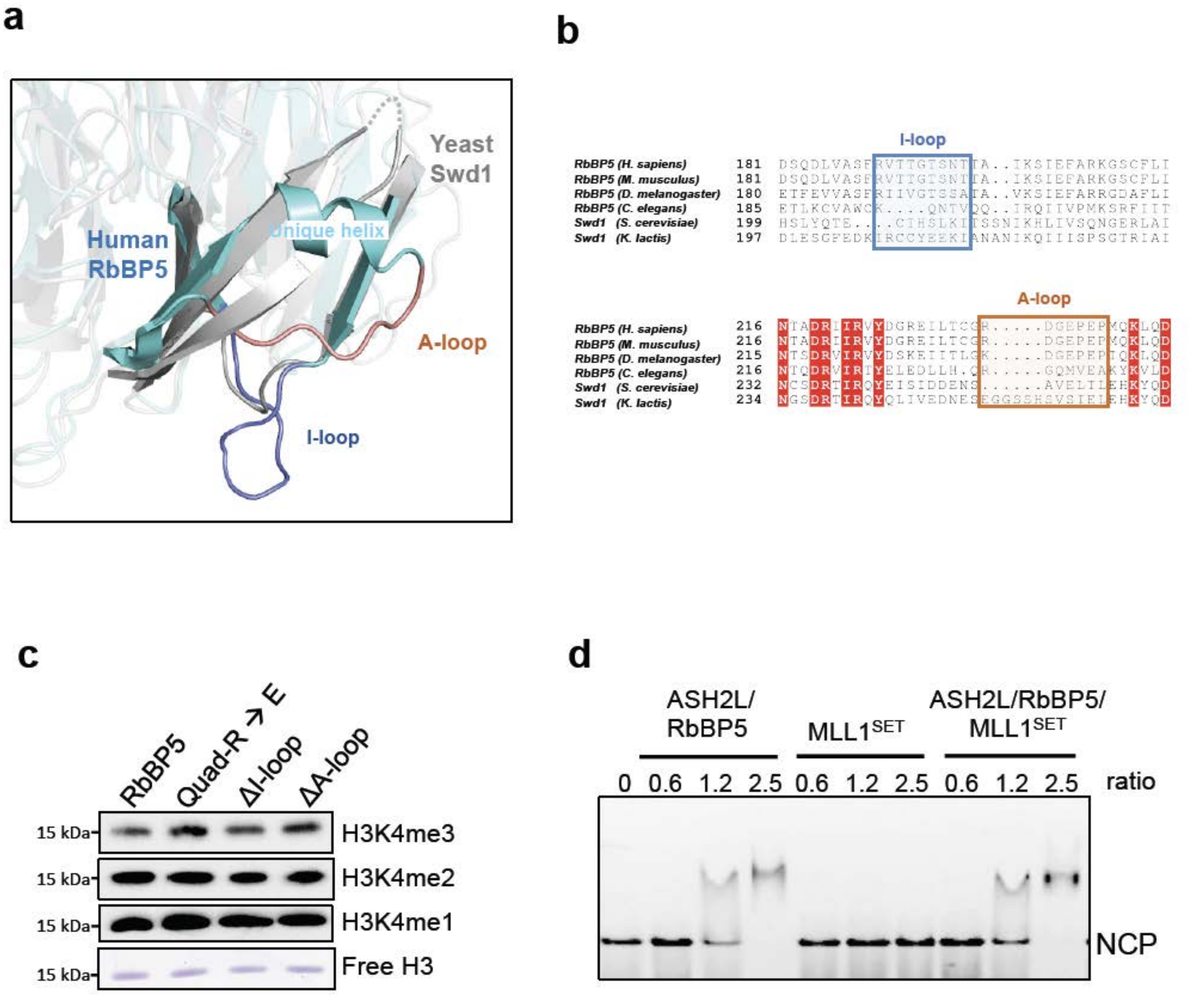

Supplementary Figure 5 | Conservation and divergence of RbBP5-NCP interaction and functional implication. **a,** Structural overlay of human RbBP5 and yeast Swd1 (PDB ID: 6CHG). RbBP5 (cyan) and Swd1 (gray) had distinct features for I- and A-loops. **b,** Multiple sequence alignment of RbBP5 I- and A-loops in eukaryotes. I- (blue box) and A- (red box) loops were highly conserved in from *D. melanogaster* to mammals. **c,** Immunoblot to detect *in vitro* histone methyltransferase activity using free histone H3 as the substrate. Reconstituted $MLL1^{RWSAD}$ complexes containing wild type and mutant RbBP5 were used, as indicated on top. Coomassie stain of H3 was included as the control. **d,** Electrophoretic mobility shift assay for the NCP with or without binding of protein or protein complexes as indicated on top. Molar ratio of protein(s) to the NCP was indicated on top.

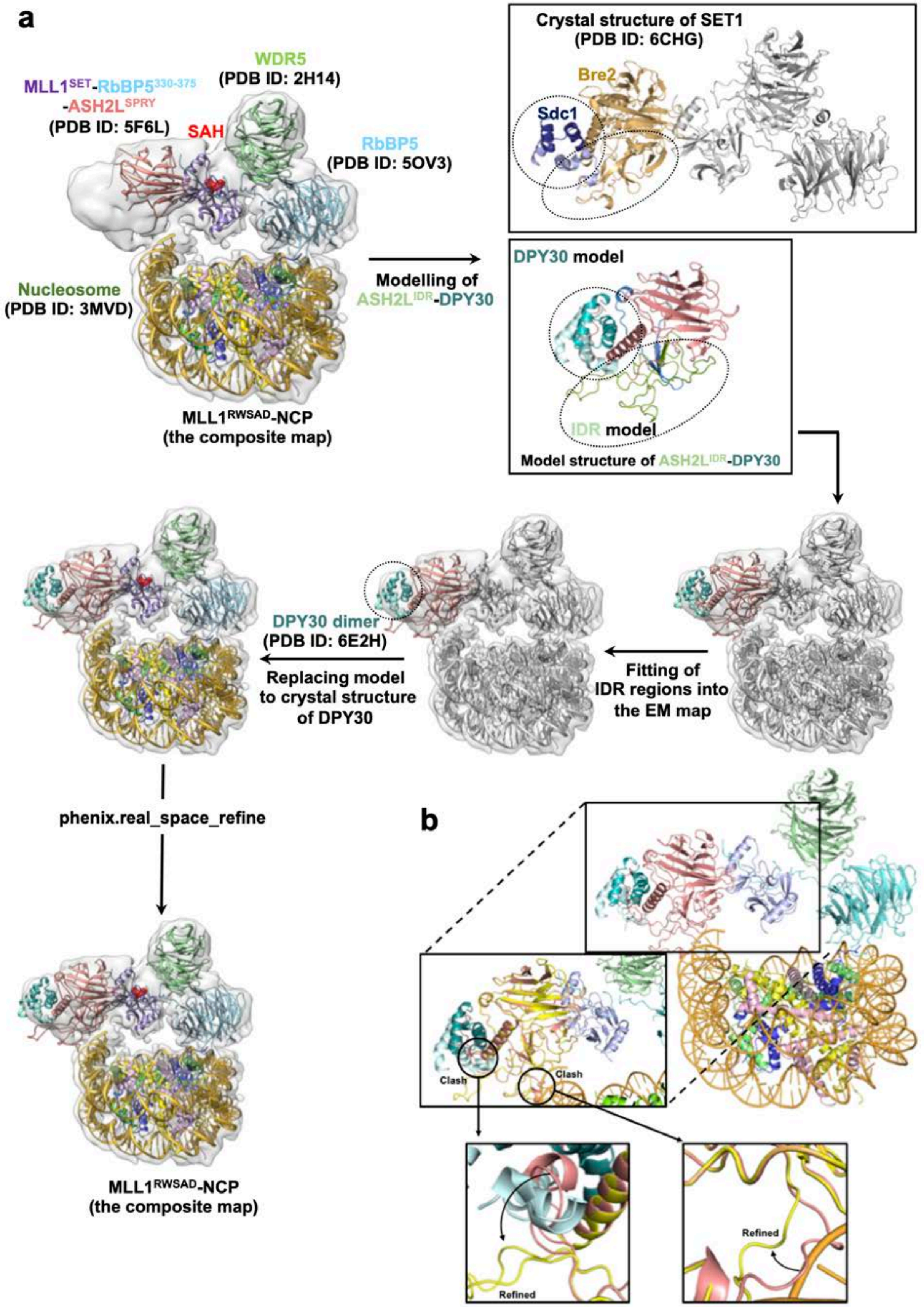

a
WDR5
(PDB ID: 2H14)
MLL1^SET-RbBP5^330-375
-ASH2L^SPRY
(PDB ID: 5F6L)
SAH
RbBP5
(PDB ID: 5OV3)
Nucleosome
(PDB ID: 3MVD)
MLL1^RWSAD-NCP
(the composite map)
Modelling of
ASH2L^IDR-DPY30
Crystal structure of SET1
(PDB ID: 6CHG)
Bre2
Sdc1
DPY30 model
IDR model
Model structure of ASH2L^IDR-DPY30
DPY30 dimer
(PDB ID: 6E2H)
Replacing model
to crystal structure
of DPY30
Fitting of
IDR regions into
the EM map
phenix.real_space_refine
MLL1^RWSAD-NCP
(the composite map)
b
Clash
Clash
Refined
Refined

Supplementary Figure 6 | Molecular modeling of the ASH2L-NCP interaction. a, Flow chart of the molecular modeling of ASH2L-IDR (also see the Methods). The crystal structure of Bre2-Sdc1 from the yeast SET1 complex (PDB ID: 6CHG) was used as template. After modeling, DPY30 dimer was replaced by crystal structure of DPY30 dimer (PDB ID: 6E2H). **b,** Fragment-guided molecular dynamic refinement to remove two clashes (black circle; pink-before, yellow-after) between ASH2L-IDR and DPY30 or DNA using the software FG-MD[3].

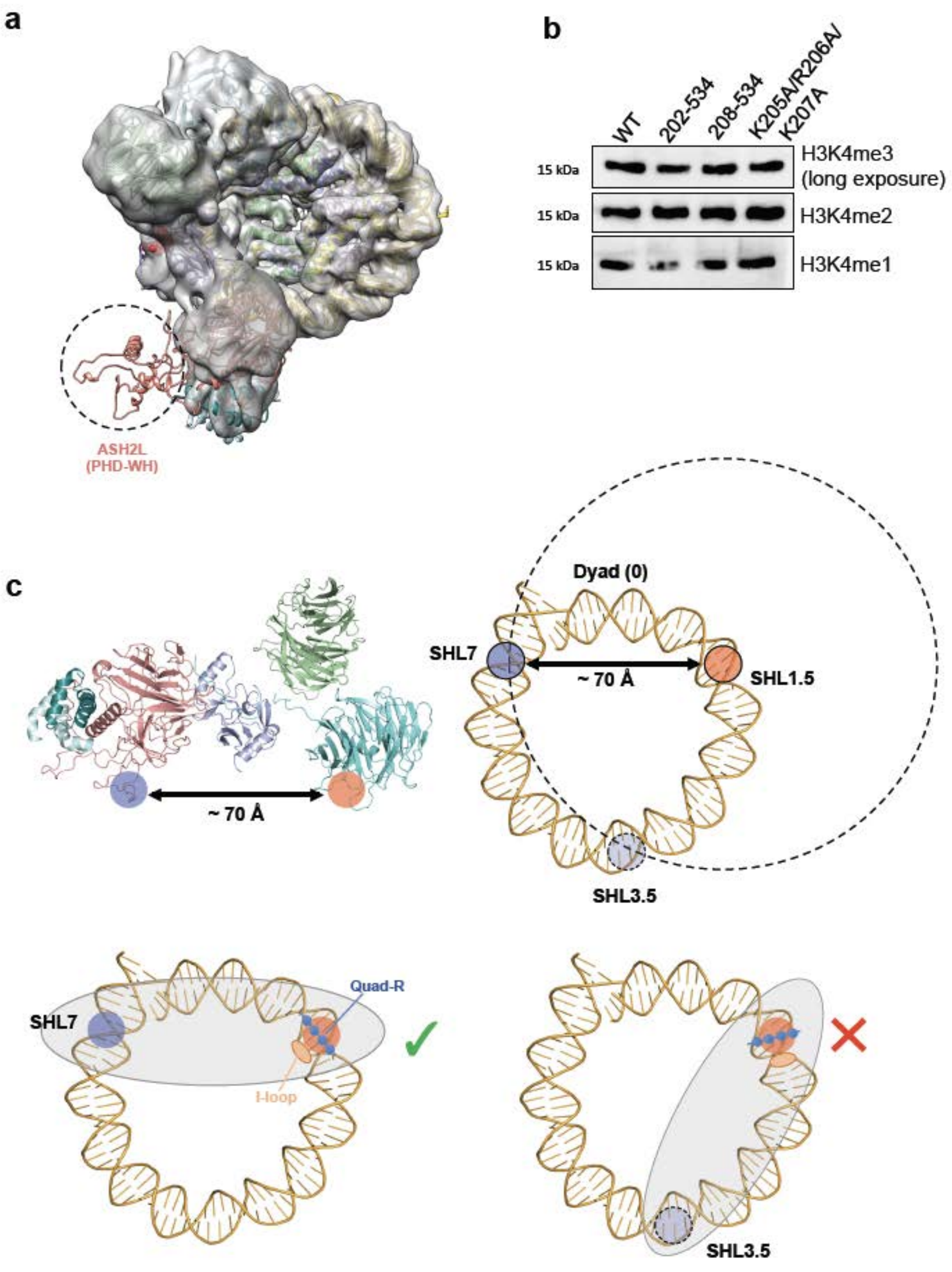

Supplementary Figure 7 | ASH2L-NCP interaction in the MLL1^{RWSAD}-NCP complex. a,

The ASH2L PHD-WH domain (model structure; black dashed circle) was not visible in the cryo-

EM map of MLL1^{RWSAD}-NCP. One potential position of the PHD-WH domain based on the structure prediction was shown. **b,** Immunoblot to detect *in vitro* histone methyltransferase activity using free histone H3 as the substrate. Reconstituted MLL1^{RWSAD} complexes containing wild type and mutant ASH2L were used, as indicated on the top. Immunoblot for RbBP5 and ASH2L were included as controls. **c,** The model for coordinated binding to the NCP by RbBP5 and ASH2L. The distance between the I-loop of RbBP5 (red circle) and the '$_{205}$-KRK-$_{207}$' basic patch of ASH2L Linker-IDR (blue circle) is ~ 70 Å. Specific anchoring of RbBP5 on the NCP confers both orientation and distance constraints for ASH2L-NCP binding. The binding of ASH2L at DNA SHL3.5 may not be allowed due to unfavorable interactions of Quad-R/DNA and I-loop/H4 tail in RbBP5-NCP (bottom right).

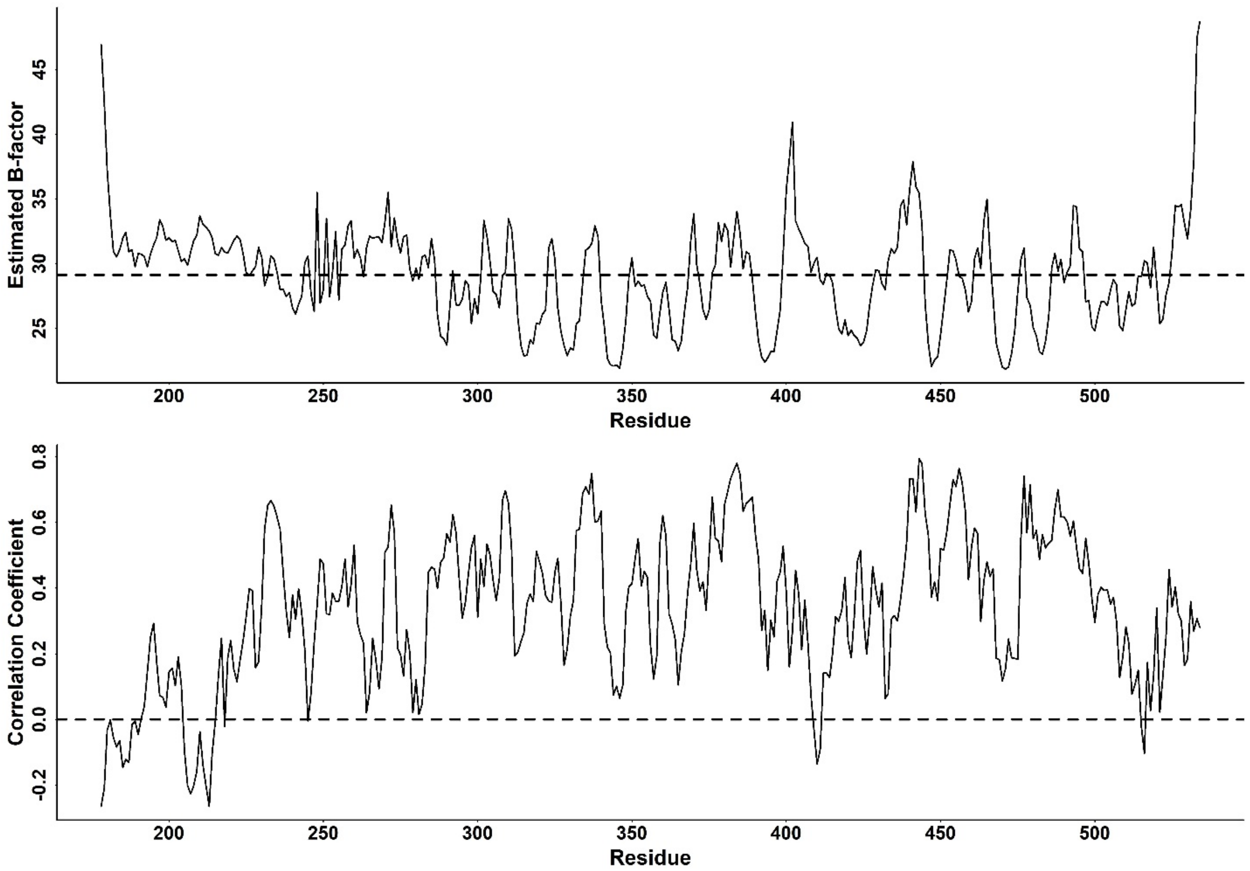

Supplementary Figure 8 | Top, residue-level estimated B-factor as the function of residues of ASH2L predicted model, where the average B-factor of the model is shown in the dash line. Bottom, correlation coefficient between the predicted model and the cryo-EM density map as the function of residues of ASH2L predicted model. Dash lines indicated the average B factor of all residues of ASH2L (top) and CC value of zero (bottom).

References

1 Kucukelbir, A., Sigworth, F. J. & Tagare, H. D. Quantifying the local resolution of cryo-EM density maps. *Nat. Methods* **11**, 63-65 (2014).

2 Afonine, P. V. *et al.* New tools for the analysis and validation of cryo-EM maps and atomic models. *Acta Crystallogr. D Struct. Biol.* **74**, 814-840 (2018).

3 Zhang, J., Liang, Y. & Zhang, Y. Atomic-Level Protein Structure Refinement Using Fragment-Guided Molecular Dynamics Conformation Sampling. *Structure* **19**, 1784-1795 (2011).
