# Supplementary material for: Cryo-EM structure of the human MLL1 core complex bound to the nucleosome
Source: Nat Commun. 2019 Dec 5;10:5540. doi: 10.1038/s41467-019-13550-2 (PMC6895043; doi:10.1038/s41467-019-13550-2)
Supplement: Supplementary file 2 — Description of Additional Supplementary Files [file 41467_2019_13550_MOESM2_ESM.docx]

**Description of Additional Supplementary Files**

**Supplementary Movie 1 | Dynamic movement of individual MLL1RWSAD subunit against the NCP.**

The movie was generated after rigid body fitting of each MLL1RWSAD subunits into 9 cryo-EM maps of MLL1RWSAD-NCP, which were shown as in Supplementary Fig. 2 (highlighted by *) and Supplementary Fig. 3e. The same color schemes in Fig. 1 were used.
