# Supplementary material for: Cryo-EM structure of the human MLL1 core complex bound to the nucleosome
Source: Nat Commun. 2019 Dec 5;10:5540. doi: 10.1038/s41467-019-13550-2 (PMC6895043; doi:10.1038/s41467-019-13550-2)
Supplement: Supplementary file 5 — Source Data [file 41467_2019_13550_MOESM5_ESM.pdf]

Figure 1B

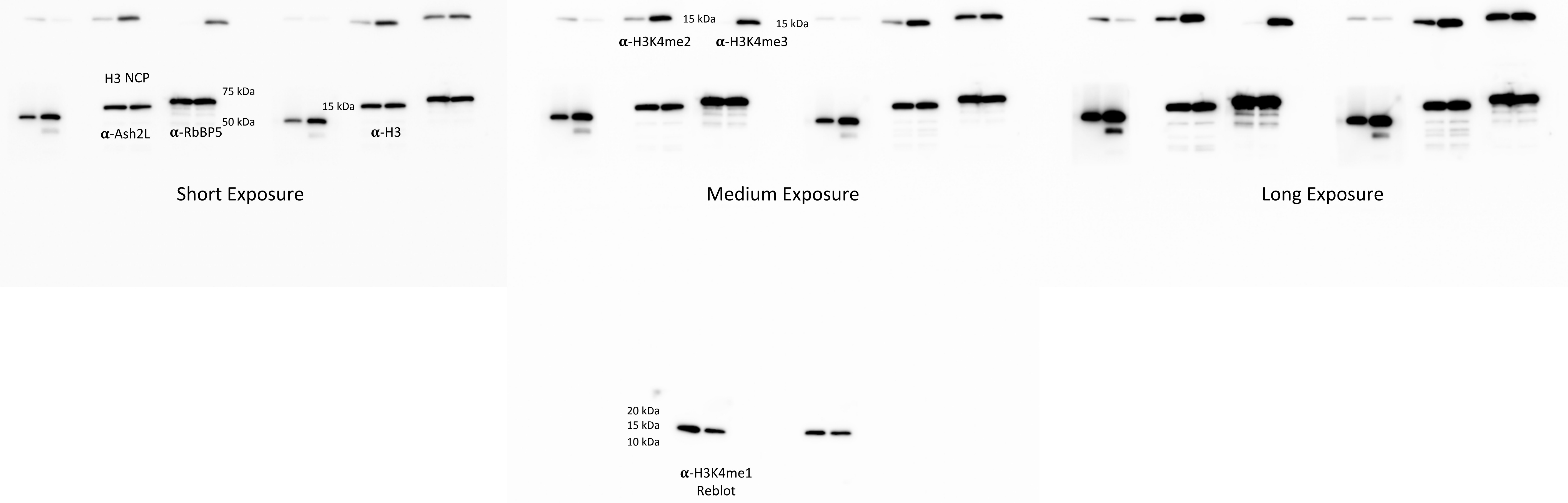

H3 NCP
75 kDa
50 kDa
15 kDa
α-Ash2L
α-RbBP5
α-H3
15 kDa
α-H3K4me2
15 kDa
α-H3K4me3
Short Exposure
Medium Exposure
Long Exposure
20 kDa
15 kDa
10 kDa
α-H3K4me1
Reblot

Figure 2C

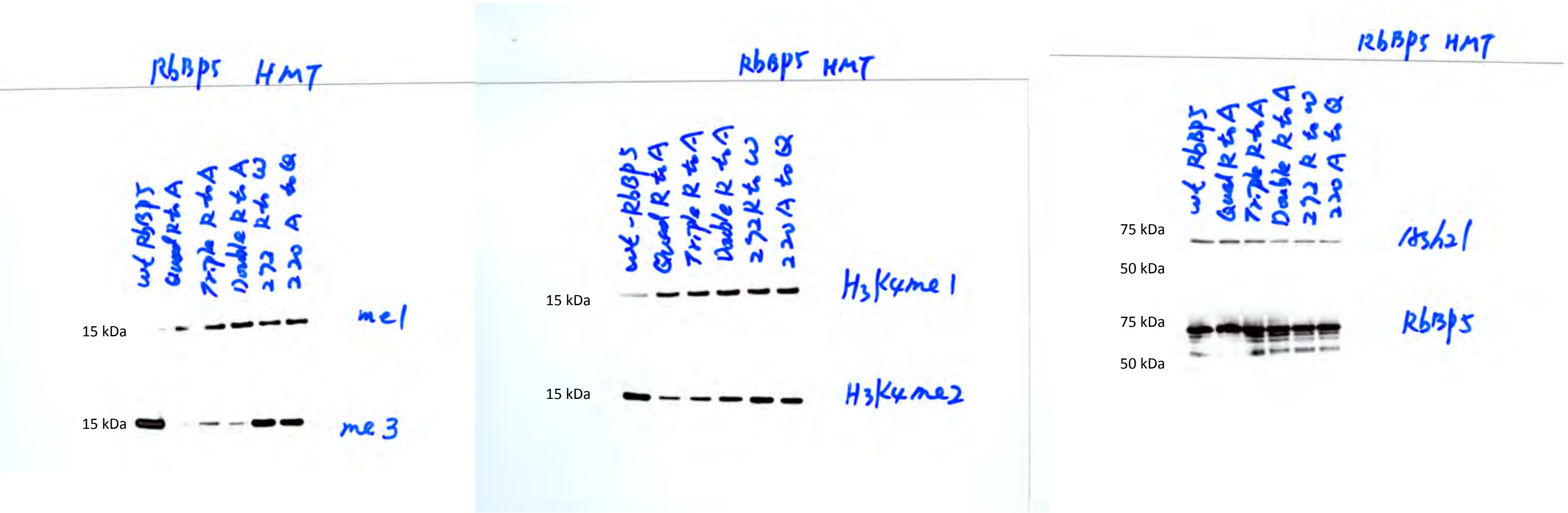
RbBP5 HMT
wt RbBP5
Quad R to A
Triple R to A
Double R to A
272 R to W
220 A to Q
15 kDa
me1
15 kDa
me3
RbBP5 HMT
wt-RbBP5
Quad R to A
Triple R to A
Double R to A
272 R to W
220 A to Q
15 kDa
H3K4me1
15 kDa
H3K4me2
RbBP5 HMT
wt RbBP5
Quad R to A
Triple R to A
Double R to A
272 R to W
220 A to Q
75 kDa
50 kDa
Ash2l
75 kDa
50 kDa
RbBP5

Figure 2D

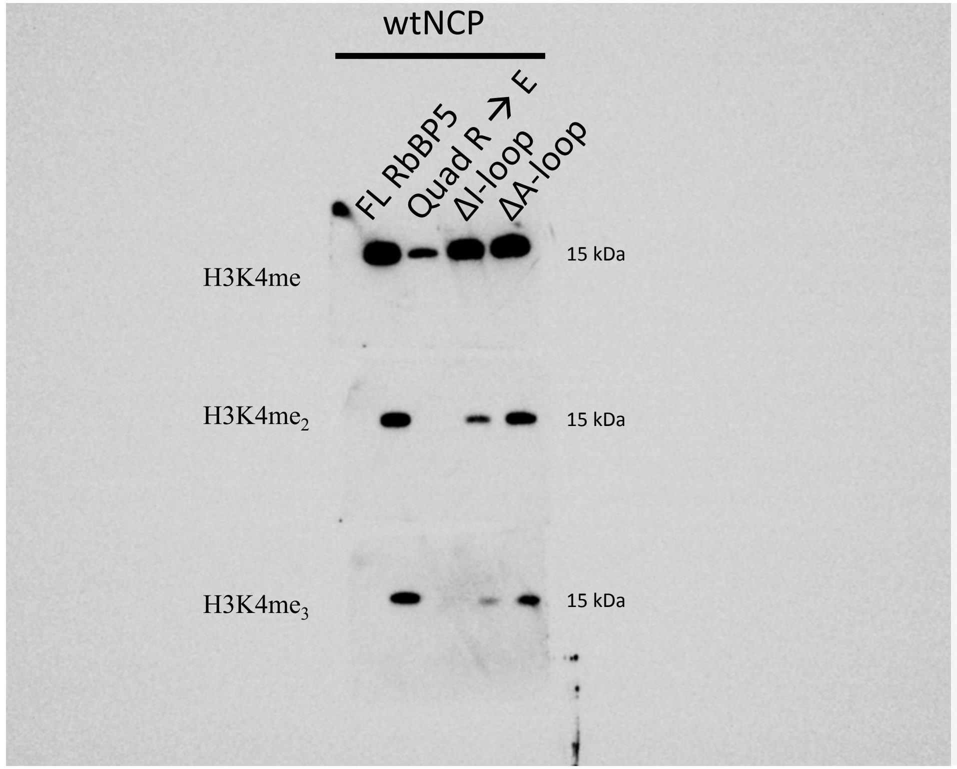

Figure 2D
RbBP5 Loading Control

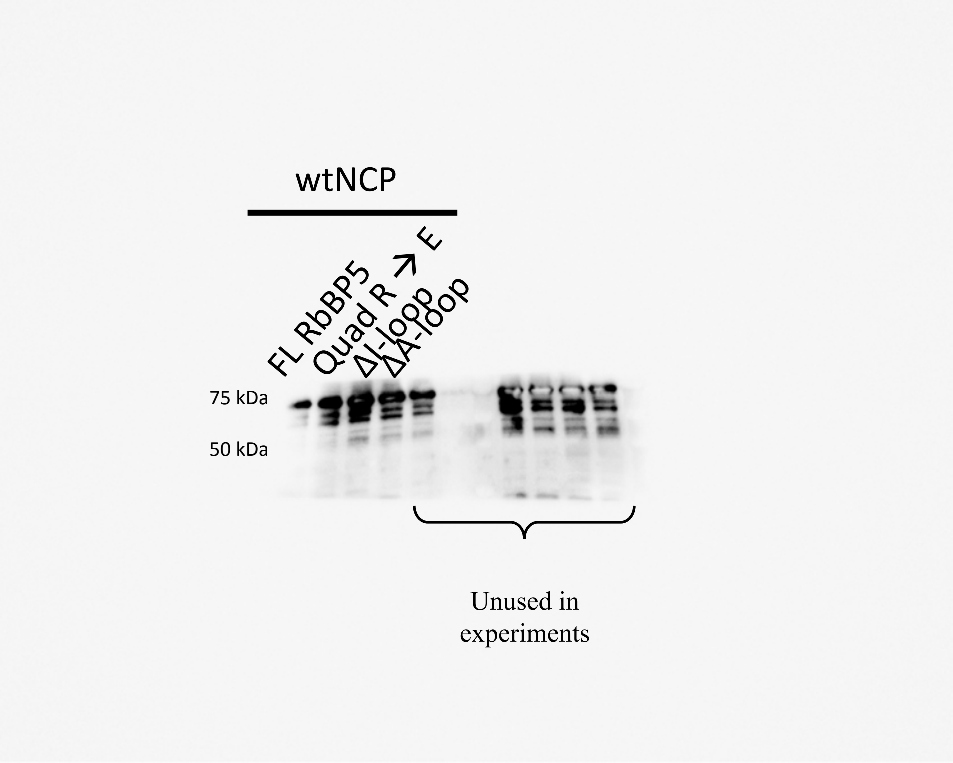

Figure 2D
Ash2L Loading Control

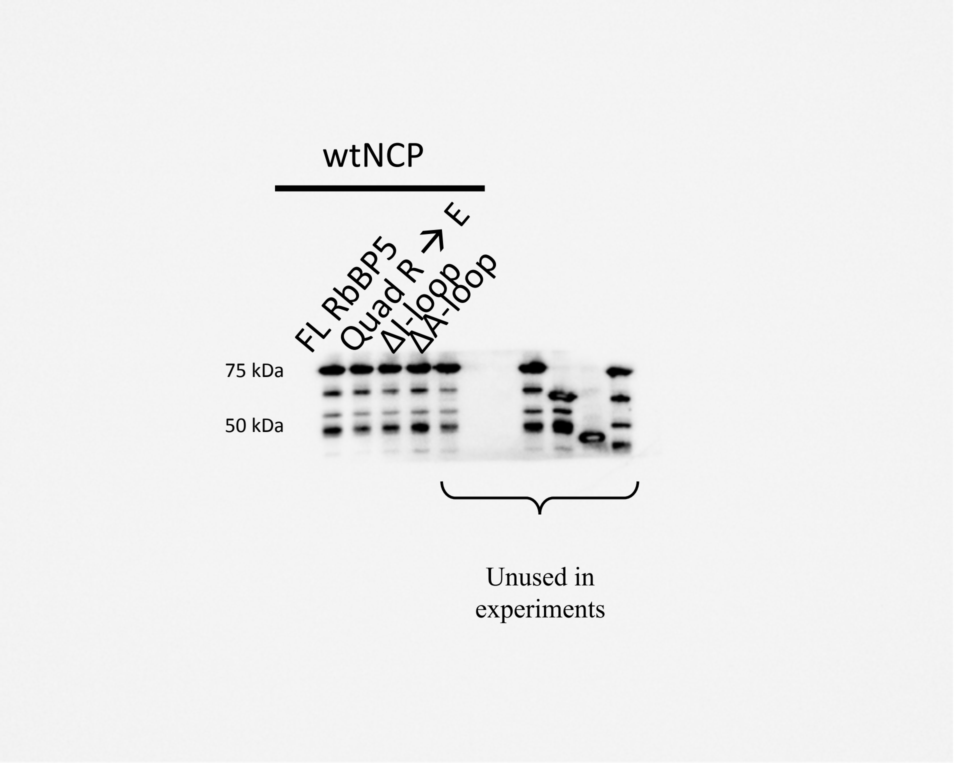

Figure 2G

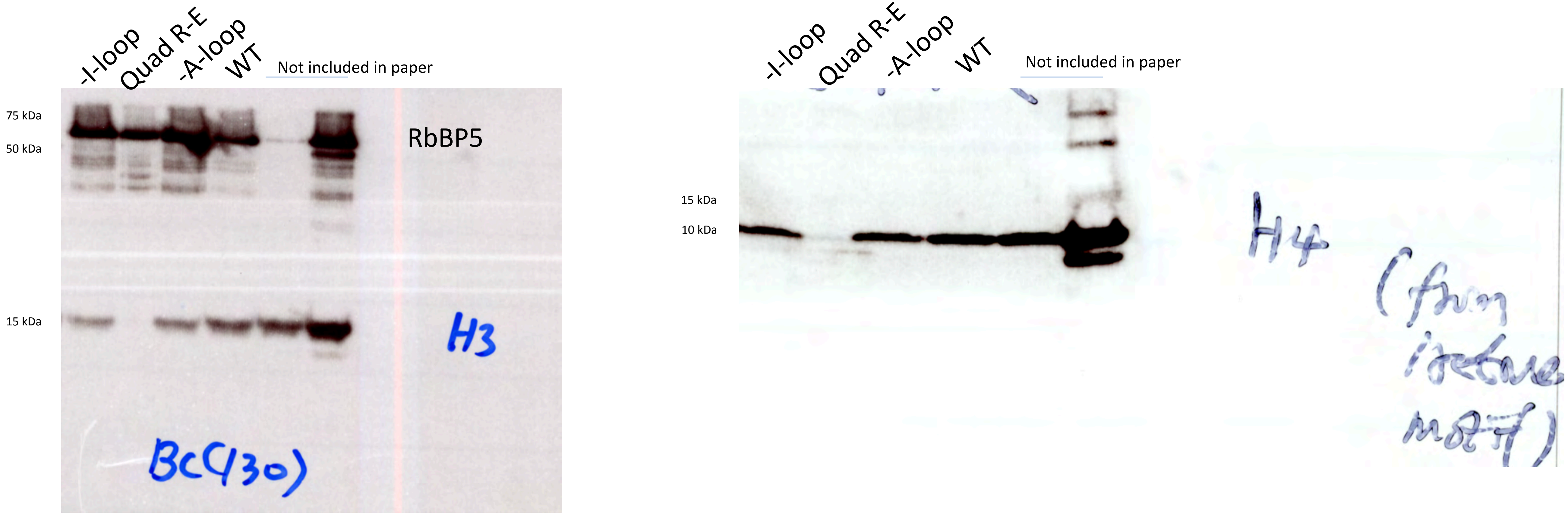
-I-loop
Quad R-E
-A-loop
WT
Not included in paper
75 kDa
50 kDa
15 kDa
RbBP5
H3
Bc(130)
-I-loop
Quad R-E
-A-loop
WT
Not included in paper
15 kDa
10 kDa
H4

Figure 5C

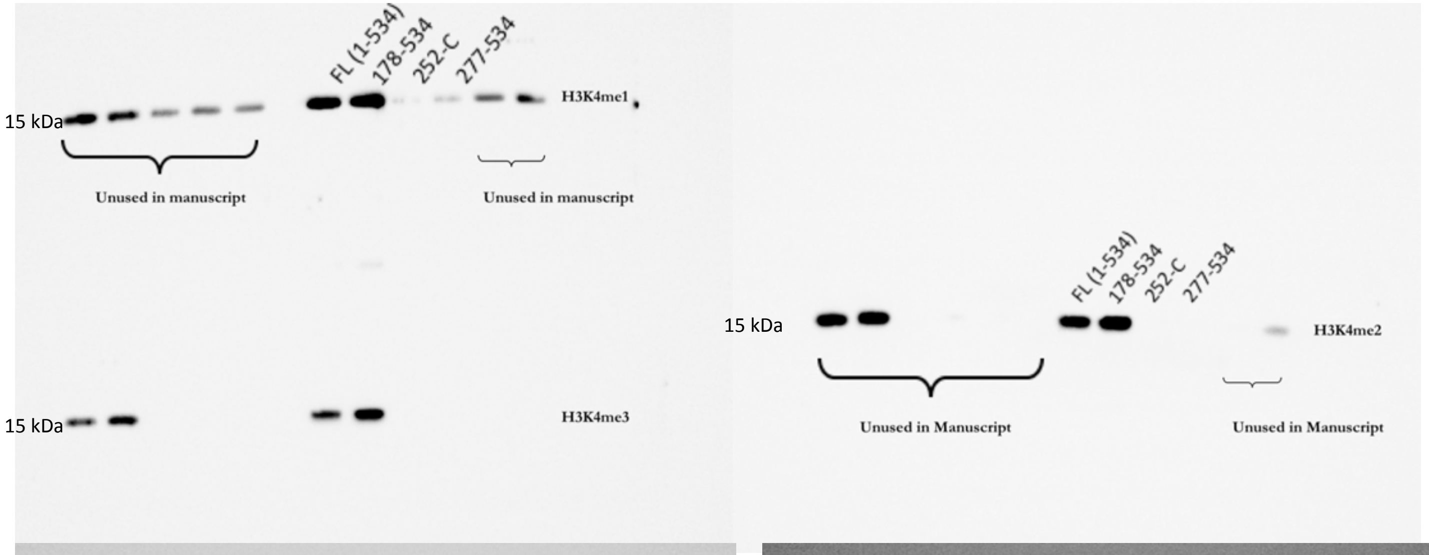
FL (1-534)
178-534
252-C
277-534
15 kDa
H3K4me1
Unused in manuscript
Unused in manuscript
15 kDa
H3K4me3
15 kDa
H3K4me2
Unused in Manuscript
Unused in Manuscript

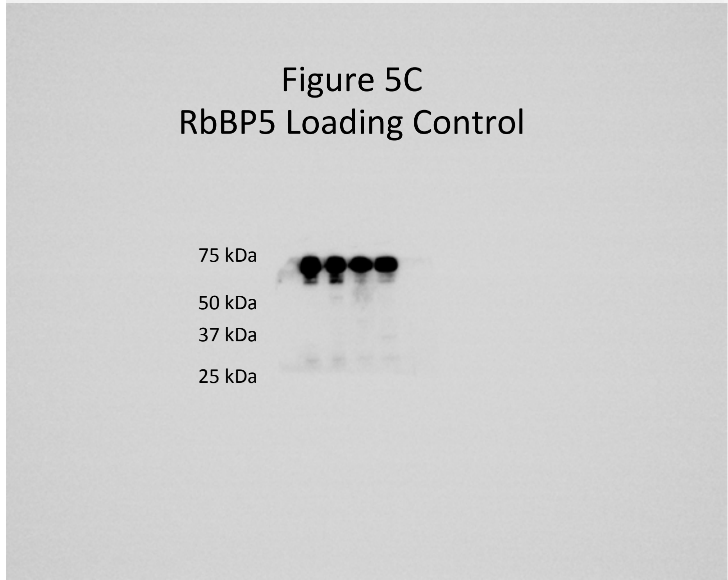
Figure 5C
RbBP5 Loading Control
75 kDa
50 kDa
37 kDa
25 kDa

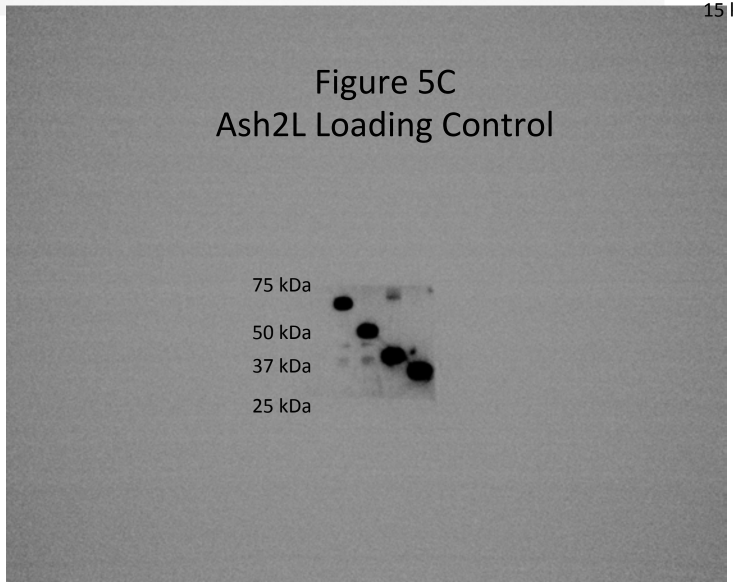
Figure 5C
Ash2L Loading Control
75 kDa
50 kDa
37 kDa
25 kDa

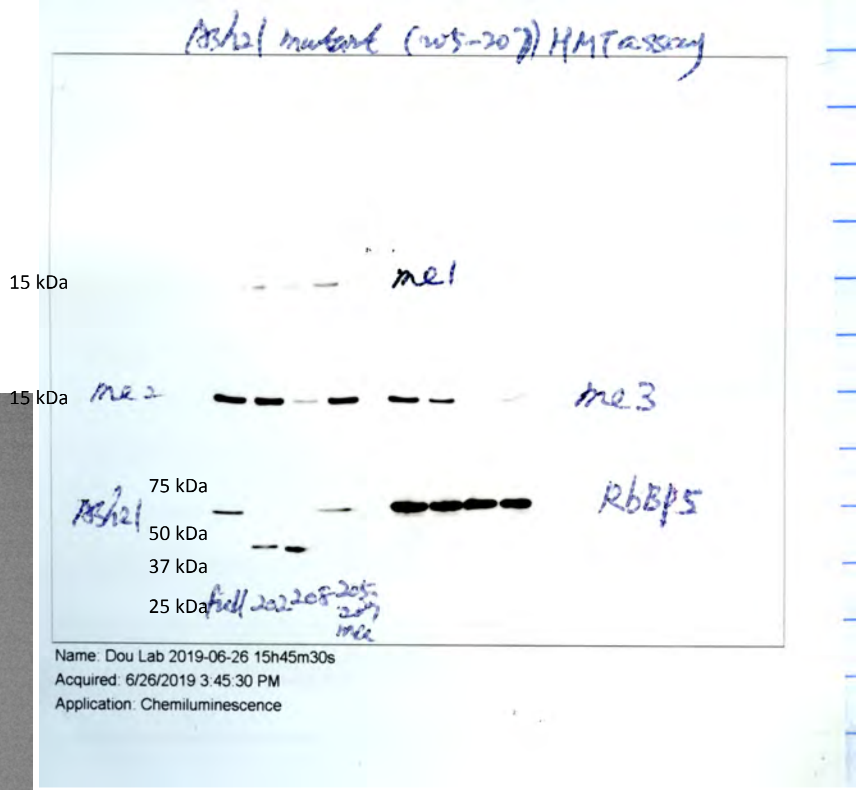
15 kDa
me1
15 kDa
me2
me3
75 kDa
RbBP5
50 kDa
37 kDa
25 kDa
Name: Dou Lab 2019-06-26 15h45m30s
Acquired: 6/26/2019 3:45:30 PM
Application: Chemiluminescence

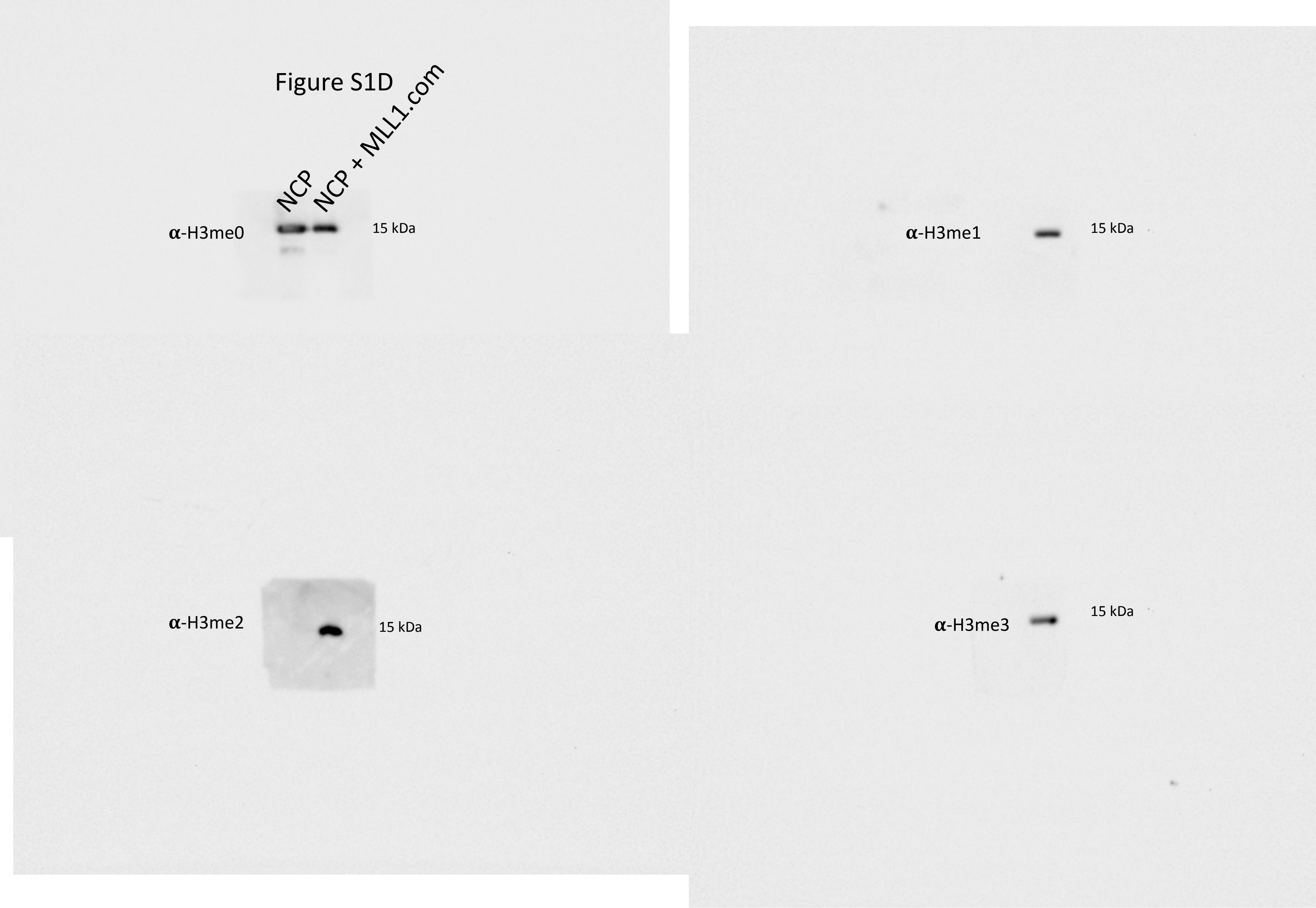
Figure S1D
NCP
NCP + MLL1.com
α-H3me0
15 kDa
α-H3me1
15 kDa
α-H3me2
15 kDa
α-H3me3
15 kDa

Figure S5C

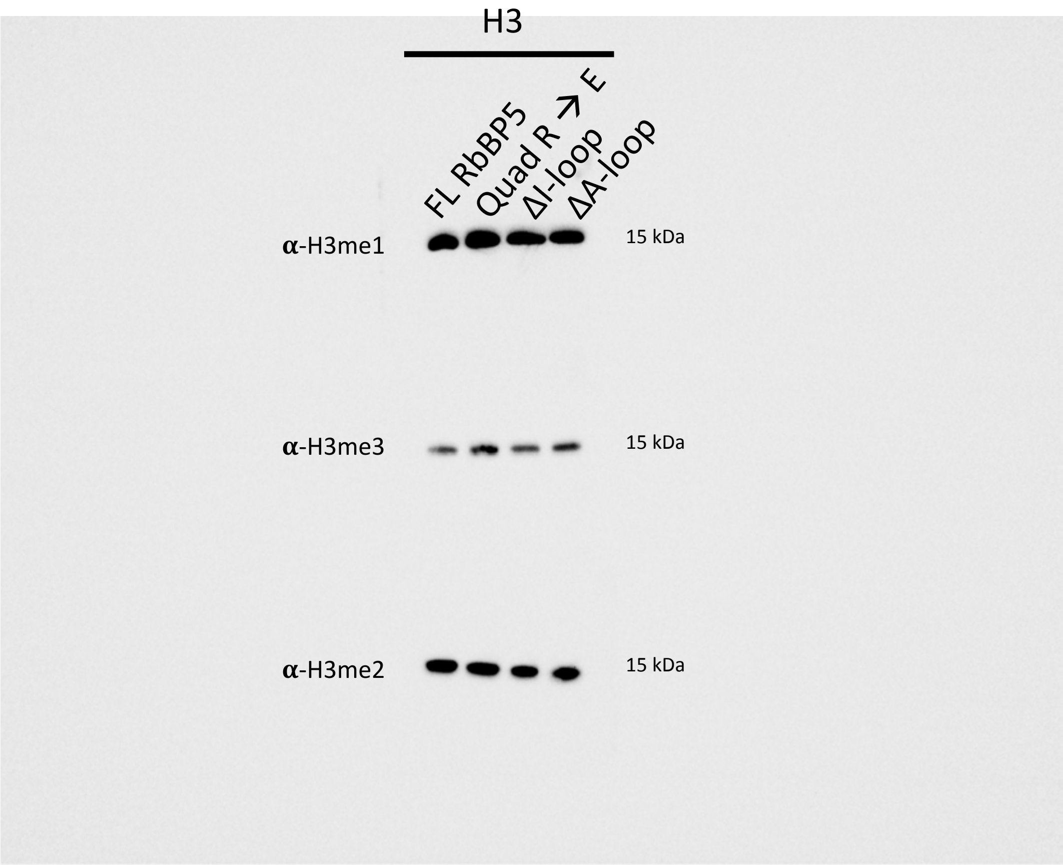

H3
FL RbBP5
Quad R → E
ΔI-loop
ΔA-loop
α-H3me1
15 kDa
α-H3me3
15 kDa
α-H3me2
15 kDa
